# Magnetic resonance-based eye tracking using deep neural networks

**DOI:** 10.1038/s41593-021-00947-w

**Published:** 2021-11-08

**Authors:** Markus Frey, Matthias Nau, Christian F. Doeller

**Affiliations:** 1 Kavli Institute for Systems Neuroscience, Centre for Neural Computation, The Egil and Pauline Braathen and Fred Kavli Centre for Cortical Microcircuits, Jebsen Centre for Alzheimer’s Disease, Norwegian University of Science and Technology, Trondheim, Norway; 2 Max Planck Institute for Human Cognitive and Brain Sciences, Leipzig, Germany; 3 Institute of Psychology, Leipzig University, Leipzig, Germany

**Keywords:** Cognitive neuroscience, Visual system, Oculomotor system, Computational neuroscience

## Abstract

Viewing behavior provides a window into many central aspects of human cognition and health, and it is an important variable of interest or confound in many functional magnetic resonance imaging (fMRI) studies. To make eye tracking freely and widely available for MRI research, we developed DeepMReye, a convolutional neural network (CNN) that decodes gaze position from the magnetic resonance signal of the eyeballs. It performs cameraless eye tracking at subimaging temporal resolution in held-out participants with little training data and across a broad range of scanning protocols. Critically, it works even in existing datasets and when the eyes are closed. Decoded eye movements explain network-wide brain activity also in regions not associated with oculomotor function. This work emphasizes the importance of eye tracking for the interpretation of fMRI results and provides an open source software solution that is widely applicable in research and clinical settings.

## Abstract (web summary)

Viewing behavior is a key variable of interest but also a confound in fMRI studies. This paper presents a deep learning framework to decode gaze position from the magnetic resonance signal of the eyeballs, which enables eye tracking in fMRI data without a camera.

## Main

Eye movements are a direct expression of our thoughts, goals and memories, and where we look determines fundamentally what we know about the visual world. The combination of eye tracking and neuroimaging can thus provide a window into many central aspects of human cognition, along with insights into neurodegenerative diseases and neural disorders of the brain^1^. A widely used tool to study human brain function is fMRI, which allows the examination of brain activity while participants engage in a broad range of tasks. Viewing behavior is either a variable of interest or one of potential confound in many fMRI studies; yet, the vast majority of them do not perform eye tracking.

We argue that eye tracking can and should be a central component of fMRI research. Not only does it allow in-depth insights into brain function but it also offers a powerful behavioral readout during scanning. Importantly, eye movements are also associated with perceptual distortions^2^, visual and motor activity^3,4^ and imaging artifacts^5^, which can severely affect the interpretation of neuroimaging results. If differences in viewing behavior between experimental conditions remain undetected, there is a high risk of misinterpreting differences in the observed brain activity^6^. Crucially, this is not restricted to studies of the visual system but affects task-based and resting-state neuroimaging on a large scale.

Magnetic resonance (MR)-compatible camera eye trackers offer a solution. They track gaze position during scanning with high temporal and spatial resolution and, hence, allow for analysis of or accounting for gaze-related brain activity. In practice, however, camera systems are being used only in a small percentage of fMRI studies. The reasons for this are manifold, but often they are simply not available or applicable in the respective research or clinical setting. Moreover, especially when viewing is not a focus of study, it may not always be obvious how the necessary investments outweigh the benefits. MR-compatible cameras are expensive, require trained staff and valuable setup and calibration time, and impose experimental constraints (for example, the eyes need to be open). Moreover, they cannot be used in blind participant groups or post hoc once the fMRI data have been acquired.

An alternative and complementary framework is MR-based eye tracking, the reconstruction of gaze position directly from the magnetic resonance signal of the eyeballs. While previous work suggested that this is indeed feasible^7–10^, several critical constraints remained that limited the usability to specific scenarios. These earlier approaches were not as accurate as required for many studies, were limited to the temporal resolution of the imaging protocol and, most importantly, required dedicated calibration scans for every single participant.

Here, we present DeepMReye, an open source cameraless eye-tracking framework based on a CNN that reconstructs viewing behavior directly from the MR signal of the eyeballs. It can be used to perform highly robust cameraless eye tracking in future fMRI experiments and also, importantly, in datasets that have already been acquired. It decodes gaze position in held-out participants at subimaging temporal resolution, performs unsupervised outlier detection and is robust across a wide range of viewing behaviors and fMRI protocols. Moreover, it can create new experimental opportunities, for example, by performing eye tracking while the eyes are closed (for example, during resting state or rapid eye movement (REM) sleep) or in groups of individuals for which eye tracker calibration remains challenging.

## Results

In the following, we present our model and results in three sections. First, we introduce our datasets, tasks, data processing pipeline and CNN in detail. Second, we show that the decoded gaze positions are highly accurate and explore the applicability and requirements of DeepMReye in depth. Third, by regressing the decoded gaze labels against the simultaneously recorded brain activity, we show that viewing behavior explains activity in a large network of regions and that DeepMReye can replace camera-based eye tracking for studying or accounting for these effects. The approach and results presented below emphasize the importance of eye tracking for MRI research and introduce a software solution that makes cameraless MR-based eye tracking widely available for free.

### Decoding gaze position from the eyeballs

**Fig. 1:**
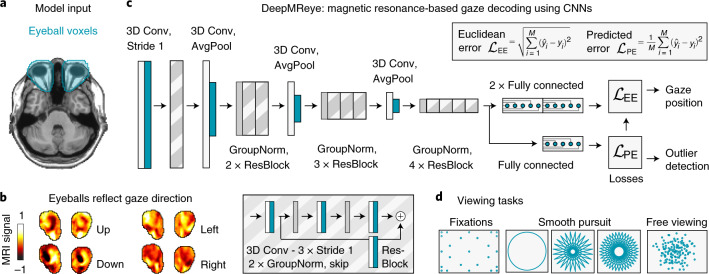
Model architecture and input. **a**, Manually delineated eye masks superimposed on a T1-weighted structural template (Colin27) at MNI coordinate *Z* = –36. **b**, Eyeball MR signal reflects gaze direction. The normalized MR signal of eye mask voxels of a sample participant who fixated on a target on the left (*X*, *Y* = –10, 0°), right (10, 0°), top (0, 5.5°) or bottom (0, –5.5°) of the screen are plotted. Source data are provided. **c**, CNN architecture. The model takes the eye mask voxels as three-dimensional (3D) input and predicts gaze position as a two-dimensional (2D; *X*, *Y*) regression target. It performs a series of 3D convolutions (3D Conv) with group normalizations (GroupNorm) and spatial downsampling via average pooling (AvgPool) in between. Residual blocks (ResBlock) comprise an additional skip connection. The model is trained across participants using a combination of two loss functions: (1) the Euclidean Error (EE) between the predicted and the true gaze position and (2) the error between the EE and a predicted error (PE). It outputs gaze position and the PE as a decoding confidence measure for each repetition time (TR). **d**, Schematics of viewing priors. We trained and tested the model on data from 268 participants performing fixations^11^, smooth pursuit on circular or star-shaped trajectories^12–14^ and free viewing^15^. Source data

We demonstrate the wide applicability of our CNN approach (Fig. 1a,b) by decoding gaze from multiple existing fMRI datasets with a total of 268 participants performing diverse viewing tasks (Fig. 1d), including fixation (dataset 1)^11^, smooth pursuit (datasets 2–4)^12–14^, visual search (dataset 5)^15^ and free picture viewing (part of dataset 6). These datasets were acquired on five 3T MRI scanners using 14 scanning protocols. Repetition times (TRs) ranged between 800 and 2,500 ms, and voxel sizes ranged between 1.5 and 2.5 mm. The eyeballs of each participant were first co-registered non-linearly to those of our group average template, which was obtained by averaging the functional images of all participants in dataset 4 (ref.^14^) fixating at the screen center. For each participant, we first aligned the head, then a facial bounding box and finally the eyeballs to the ones of our template. This optional three-step procedure ensured that the eyeballs were aligned across participants and that the average gaze position reflected the screen center (Supplementary Fig. 1). Note that potential offsets to the screen center can be estimated in the training data and then factored in after the decoding. The template brain has itself been co-registered to a Montreal Neurological Institute (MNI) structural template in which the eyes were manually segmented (Fig. 1a). We then extracted the multivoxel pattern (MVP) of the eyes at each imaging acquisition, normalized the pattern in time and space (Fig. 1b) and fed it into the CNN (Fig. 1c). While the exact model training and test procedure will be explained in detail later, it essentially uses the MVP of the eyes to predict ten on-screen gaze coordinates corresponding to the respective volume. For the main analyses, these ten gaze labels per TR were obtained either using camera-based eye tracking in the case of the unconstrained visual search dataset^15^ or from the screen coordinates of the fixation target in the case of all others^11–14^. For the final model evaluation, these ten gaze labels were median averaged to obtain one gaze position per TR. The CNN was trained using cross-validation and a combination of two weighted loss functions (Fig. 1c), (1) the ‘Euclidean error’ (EE) between real and predicted gaze position and (2) a ‘predicted error’ (PE). The latter represents an unsupervised measure of the expected EE given the current input data.

### Decoding viewing behavior in held-out participants

**Fig. 2:**
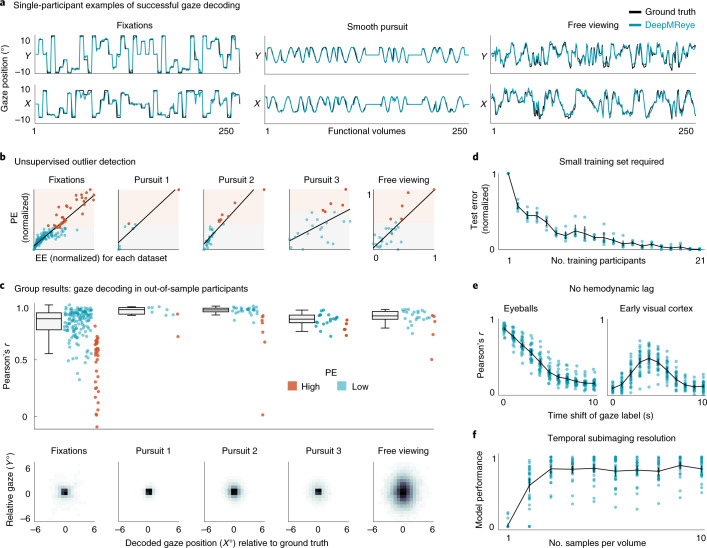
Across-participant gaze decoding results. **a**, Single-participant examples of successful gaze decoding for three viewing behaviors. **b**, Predicted error (PE) correlates with the Euclidean error (EE) between real and predicted gaze positions. This allows for filtering of the test set after decoding based on estimated reliability. Single-participant data with a regression line are plotted. Participants were split into 80% most reliable (low PE, blue) and 20% least reliable participants (high PE, orange). Scores are normalized for visualization. **c**, Group results. Top, gaze decoding expressed as the Pearson correlation between true and decoded gaze trajectory for the five key datasets featuring fixations, 3× smooth pursuit and visual search. Participants are color coded according to PE. Whisker box plots for low-PE participants (central line, median; box, 25th and 75th percentile; whiskers, all data points not considered outliers; outliers, data points outside 1.5× interquartile range) and single-participant data (blue and orange dots) for all are plotted. Bottom, time-collapsed group average histograms of decoded positions relative to the true positions (0, 0) in visual degrees. The color depicts decoding probability (black represents high). **d**, Test error as a function of how many participants were used for model training. **e**, Gaze decoding from the eyeballs and early visual cortex for time-shifted gaze labels. **f**, Subimaging temporal resolution. The model performance (explained variance normalized for each participant) depending on how many subimaging samples were decoded is plotted. The data in **d**–**f** show group-level mean ± s.e.m. (black lines) as well as single-participant data (blue dots) of the results obtained for the visual search dataset 5. Source data are provided. Source data

**Extended Data Fig. 1:**
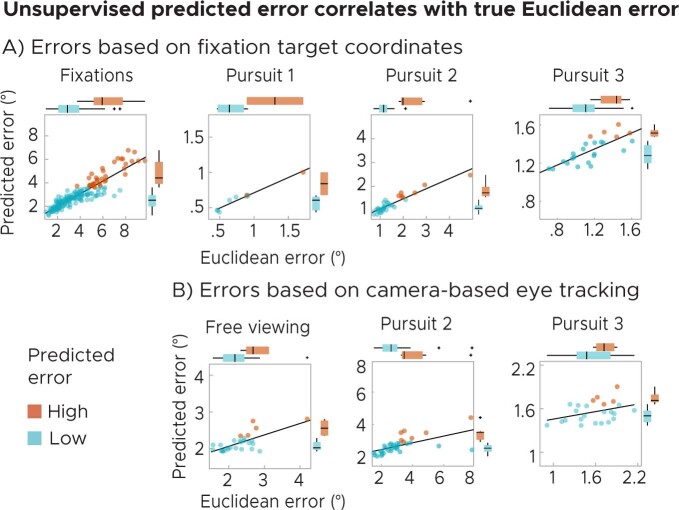
Predicted error (PE) correlates with the Euclidean error between real and predicted gaze positions. This allows to filter the test set post-decoding based on estimated reliability. A) Results plotted for models trained and tested using the fixation target coordinates. B) Results plotted for models trained and tested using labels acquired using camera-based eye tracking. A, B) We plot single-participant data (dots) with regression line as well group-level Whisker-box-plots (central line: median, box: 25th and 75th percentile, whisker: all data points not considered outliers, outliers: data points outside 1.5x interquartile range). Participants were split into 80% most reliable (Low PE, blue) and 20% least reliable participants (high PE, orange). All scores expressed in visual degrees. Source data

**Extended Data Fig. 2:**
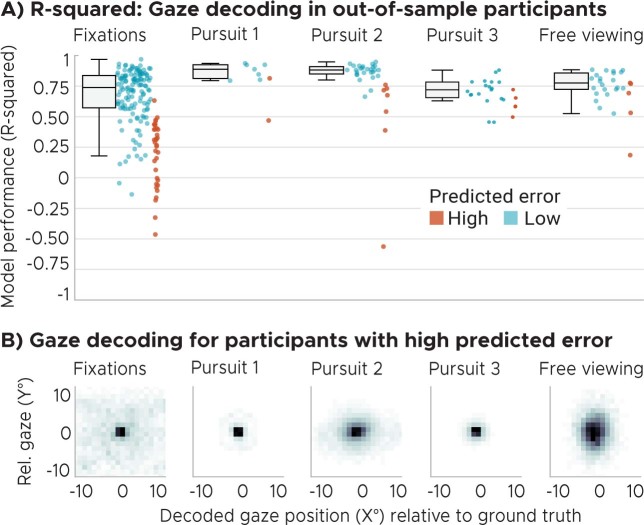
Quantifying gaze decoding in high predicted error and out-of-sample participants. A) Gaze decoding group results expressed as the coefficient-of-determination (*R*^2^). Top panel shows gaze decoding expressed as the *R*^2^-score implemented in scikit-learn^40^ between the true and decoded gaze trajectory for the five key datasets featuring fixations, 3x smooth pursuit and visual search. Note that *R*^2^ can range from negative infinity to one. Participants are color coded according to predicted error (PE). We plot Whisker-box-plots for Low-PE participants (central line: median, box: 25th and 75th percentile, whisker: all data points not considered outliers, outliers: data points outside 1.5x interquartile range) and single-participant data for all (dots). (B) Group-average spread of decoded positions around true positions collapsed over time in visual degrees for participants with high predicted error (orange dots in A). Source data

**Extended Data Fig. 3:**
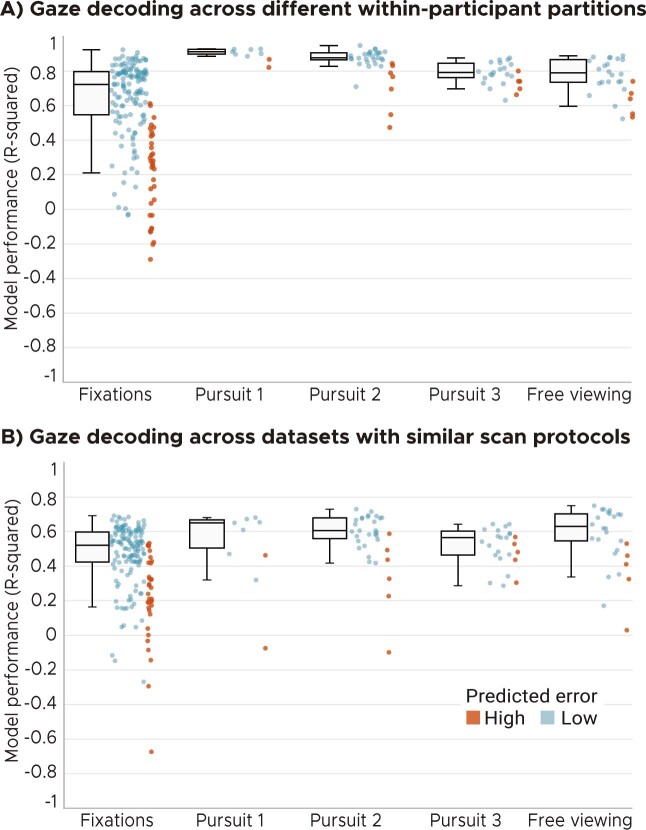
Model evaluation across different decoding schemes. A) Within-participant gaze decoding obtained by training and testing the model on different data partitions of all participants within a dataset. B) Across-dataset gaze decoding obtained using leave-one-data-set-out cross-validation. We plot the *R*^2^-score as implemented in scikit-learn^40^ between true and decoded gaze trajectory for the five key datasets featuring fixations, 3x smooth pursuit and visual search. Note that *R*^2^ can range from negative infinity to one. The results of datasets 1-3 were obtained using the fixation target labels, the ones of datasets 4-5 were obtained using camera-based eye tracking labels. Participants are color coded according to predicted error (PE). A, B) We plot Whisker-box-plots for Low-PE participants (central line: median, box: 25th and 75th percentile, whisker: all data points not considered outliers, outliers: data points outside 1.5x interquartile range) and single-participant data for all (dots). Source data

First, we examined the decoding performance at image-wise resolution in the five key datasets that were acquired for other purposes (datasets 1–5 (refs. ^11–15^); Methods and Fig. 2). The model was trained and tested using an across-participant decoding scheme, meaning that it was trained on 80% of the participants within each dataset and then tested on the held-out 20% of participants of that dataset. This procedure was cross-validated until all participants were tested once. For all viewing behaviors, we found that the decoded gaze path followed the ground truth gaze path closely in the majority of participants (Fig. 2a). To quantify gaze decoding on the group level, we computed four measures: the EE (Fig. 2b and Extended Data Fig. 1), the Pearson correlation (*r*; Fig. 2c), the coefficient of determination (*R*^2^; Extended Data Fig. 2a) between the real and the decoded gaze paths of each participant and the error as a fraction of total stimulus size (FoS). We found that gaze decoding worked in the large majority of participants with high precision (Fig. 2c and Extended Data Fig. 2b) and for all viewing behaviors tested (median performance of the 80% most reliable participants (low PE): all datasets, *r* = 0.89, *R*^2^ = 0.78, EE = 1.14°, FoS = 7.6%; fixation, *r* = 0.86, *R*^2^ = 0.74, EE = 2.89°, FoS = 11%; pursuit 1, *r* = 0.94, *R*^2^ = 0.89, EE = 0.64°, FoS = 5%; pursuit 2, *r* = 0.94, *R*^2^ = 0.88, EE = 1.14°, FoS = 8%; pursuit 3, *r* = 0.86, *R*^2^ = 0.72, EE = 1.11°, FoS = 5%; free viewing, *r* = 0.89, *R*^2^ = 0.78, EE = 2.17°, FoS = 9%). These results were also robust when independent data partitions of each participant were used for training and testing (within-participant decoding scheme, Extended Data Fig. 3a), and we uncovered gaze position even when independent datasets were used for model training and testing (across-dataset decoding, Extended Data Fig. 3b). Moreover, by shuffling the time courses of individual voxels and quantifying the influence on the EE of our model, we further found that the information used for decoding indeed originated mostly in the eyeballs and the optic nerves (Supplementary Fig. 2). Together, these results demonstrate that gaze decoding with DeepMReye can be highly reliable and accurate. It allows reconstructing even complex viewing behaviors in held-out participants, critically relying solely on the MR signal of the eyeballs without requiring any MR-compatible camera equipment.

### Unsupervised outlier detection

**Extended Data Fig. 4:**
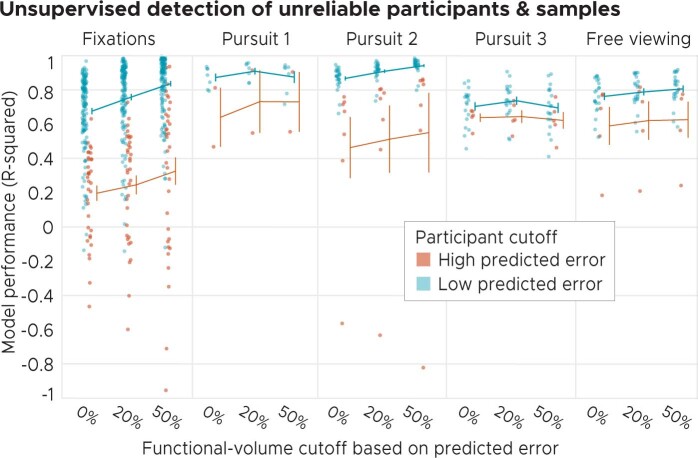
Model performance evaluated before and after exclusion of volumes with unreliable decoding. Here, before computing model performance we filtered out either the 0%, 20% or 50% least reliable volumes (that is those with the highest predicted error (PE)). Model performance is expressed as the coefficient-of-determination *R*^2^-score implemented in scikit-learn^40^ between true and decoded gaze trajectory for the five key datasets featuring fixations, 3x smooth pursuit and visual search. Note that *R*^2^ can range from negative infinity to one. We plot single-participant data (dots) as well as the mean ± standard error of the mean (line plots). Participant dots were additionally color coded according to the participants’ PE. Source data

As mentioned above, the model computes a PE score for each sample and participant in addition to decoding gaze position. Importantly, this PE correlated with the true EE across participants, allowing for the detection of participants for which the decoding did not work as well as for others (Fig. 2b and Extended Data Fig. 1a,b). For example, if the eye masks were not well co-registered, if an eye was missing or if there were other sources of noise, the voxel values obtained for the respective participant would differ from those of other participants. Accordingly, the outlier participant would show a high EE because the voxels would not be informative about the gaze position. The model learns to recognize these features in the data associated with accurate and inaccurate decoding, and it expresses its own estimate of decoding reliability in the form of the PE. This relative measure of decoding reliability can thus be used to remove outliers from subsequent analysis or to account for them, for example, by adding covariate regressors in group analyses. Moreover, in addition to detecting outlier participants, the PE also allowed for removal of outlier samples within each participant, which further improved the reliability of the results (Extended Data Fig. 4). Note that in addition to computing the PE, our pipeline visualizes various features of the input data (Supplementary Fig. 3), which can further aid quality assessment.

### No camera required for model training

**Extended Data Fig. 5:**
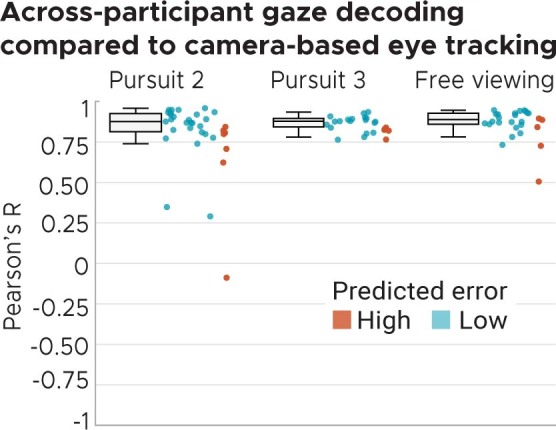
Gaze decoding evaluated using camera-based eye tracking for smooth pursuit datasets 3-4. Model performance expressed as the Pearson correlation between true and decoded gaze trajectory for the datasets with camera-based eye tracking. Because the visual search dataset 5 used labels obtained using camera-based eye tracking as well, we additionally plot the results obtained for this dataset again for the sake of completeness. Participants are color coded according to predicted error (PE). We plot Whisker-box-plots for Low-PE participants (central line: median, box: 25th and 75th percentile, whisker: all data points not considered outliers, outliers: data points outside 1.5x interquartile range) and single-participant data for all (dots). Source data

We next explored our model’s requirements and boundary conditions in detail. First, we tested what type of training labels are required for DeepMReye, finding that both the screen coordinates of a fixation target (Fig. 2c) and labels obtained using camera-based eye tracking (Extended Data Fig. 5) led to similar performance. While the results presented for dataset 5 (Fig. 2c) already reflect the results obtained with camera-based labels, we additionally reran the model on gaze labels obtained via camera-based eye tracking for the smooth pursuit datasets 3 and 4 (Extended Data Fig. 5). Thus, because DeepMReye can be trained on fixation target labels only and because it generalizes across participants (Fig. 2), users could acquire fMRI data for a few participants performing various fixation tasks, record the screen coordinates of the fixation target as training labels, train the model on these labels and then decode from all other participants. We provide the code for an experimental paradigm that can be used to produce such training labels (see Code availability and online user documentation).

### Small training set

**Extended Data Fig. 6:**
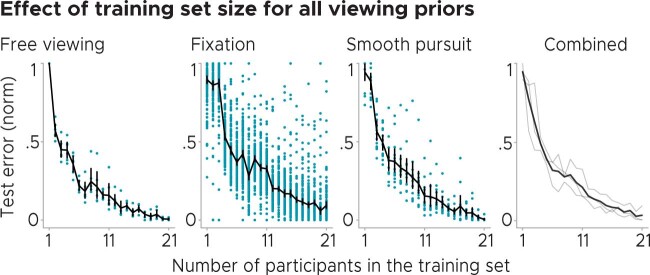
Normalized test error as a function of how many participants were used for model training plotted for three different viewing behaviors. We plot single participant data (dots) as well as the across-participant average model performance (black lines). Error bars depict the standard error of the mean. Right panel shows the average across datasets. Source data

**Extended Data Fig. 7:**
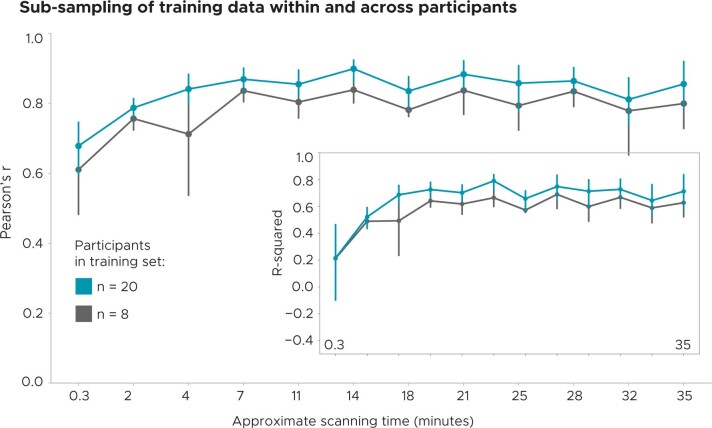
Across-participant decoding performance as a function of how much single-participant data was considered for model training as well as of the number of participants in the training data (n=8 and n=20). We plot the group-level mean (line plots) ± standard error of the mean (error bars) of the model performance expressed as the Pearson correlation and the R-squared score between real and predicted gaze path in the test set. For free viewing, model performance saturates at as little as 5-10 Minutes of training data. Note that these results likely depend on the viewing behavior and on how similar the behavior is across data partitions and participants. Source data

Next, we asked how many participants were required for model training as well as how much data are needed to be acquired for each one. We tested this by iteratively subsampling the number of participants in the training set, each time testing how well the model performed on the same test participants. We chose to conduct this analysis on dataset 5 because it featured the most natural and hence most complex viewing pattern tested. We found that model performance improved with an increasing training set size but also that model performance approached the ceiling level at as few as six to eight participants (mean performance: one participant, *r* = 0.43, *R*^2^ = 0.11, EE = 5.12°; five participants, *r* = 0.81, *R*^2^ = 0.62, EE = 3.18°; ten participants, *r* = 0.86, *R*^2^ = 0.71, EE = 2.58°; Fig. 2d and Extended Data Fig. 6). We then repeated this analysis while subsampling the amount of single-participant data considered during training in addition to the number of participants in the training set. We found that model performance saturated at as little as 5 min worth of free-viewing data (Extended Data Fig. 7). This suggests that even a small training set can yield a well-trained model and hence reliable decoding results. Note, however, that model performance likely also depends on how similar the expected viewing behavior is between training and test sets. If the gaze pattern is very similar across participants, which can be the case even for viewing of complex stimuli, such as real-world scenes^16^, decoding it in independent participants can work even better despite a small training set. This fact can be seen, for example, in our main results for the smooth pursuit dataset 2 (ref. ^12^) (Fig. 2).

### No hemodynamic component

Naturally, when the eyes move, the surrounding tissue undergoes dramatic structural changes, which are expected to affect the MR signal acquired at that time. To test whether this is the source of information used for decoding, we shifted the gaze labels relative to the imaging data by various TRs (0–10), each time training and testing the model anew. Indeed, we found that the eyeball decoding was most accurate for the instantaneous gaze position and that no hemodynamic factors needed to be considered (Fig. 2e). This is in stark contrast to decoding from brain activity for which the same model pipeline can be used (Fig. 2e). In the primary visual cortex (V1), decoding was optimal after around 5–6 s (*r* = 0.483 ± 0.132) and followed the shape of the hemodynamic response function.

### Subimaging temporal resolution

**Extended Data Fig. 8:**
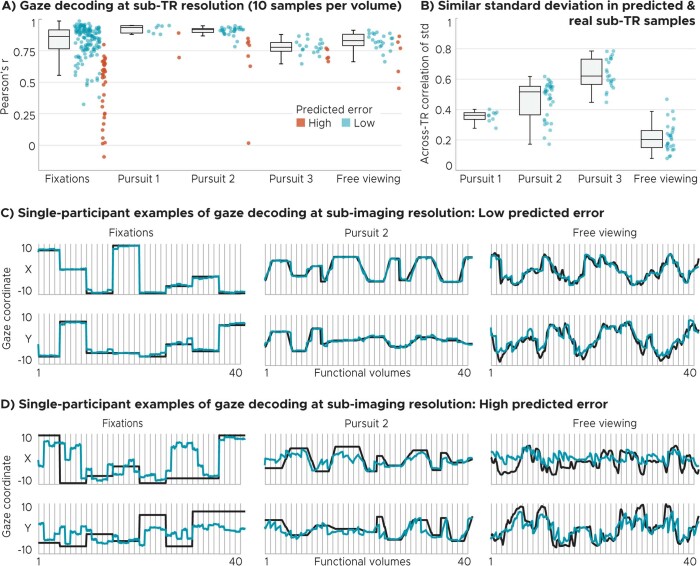
Sub-imaging decoding resolution. A) Group results when all 10 sub-TR samples are considered for computing the Pearson correlation between true and decoded gaze trajectories. Participants are color coded according to predicted error (PE). We plot Whisker-box-plots for Low-PE participants (central line: median, box: 25th and 75th percentile, whisker: all data points not considered outliers, outliers: data points outside 1.5x interquartile range) and single-participant data for all (dots). B) Similar standard deviation of real and decoded gaze labels within each functional volume (TR), that is if the 10 real gaze labels of a TR had a high standard deviation (indicating larger eye movements within this TR) then the 10 decoded gaze labels showed a high standard deviation as well. We plot the Pearson correlation between the within-TR standard deviation computed using the full time course of each participant as Whisker-box-plots (central line: median, box: 25th and 75th percentile, whisker: all data points not considered outliers, outliers: data points outside 1.5x interquartile range) and single-participant data as dots. C, D) Single-participant examples of gaze decoding at a virtual sub-imaging resolution of 10 samples per volume. We plot three example participants with low predicted error (C) and three example participants with high predicted error (D) for the fixation, smooth pursuit and free-viewing datasets^11,14,15^. Functional-volume onsets plotted as grey vertical lines. Source data

The results presented so far were obtained by decoding the average gaze position from each volume in independent data. We believe this image-wise-resolution eye tracking already enables a multitude of exciting applications as discussed below. Intriguingly, however, because different imaging slices were acquired at different times and because the MR signal of a voxel can be affected by motion, it should, in principle, be possible to decode gaze position at a temporal resolution higher than the one of the imaging protocol (sub-TR resolution). As mentioned above, DeepMReye classifies ten gaze labels per functional volume, which are median averaged to obtain one gaze position per TR. This procedure yielded a higher decoding performance than classifying only one position, and it enabled testing of how well the gaze path can be explained by the sub-TR labels themselves (Extended Data Fig. 8a). We found that during visual search, more gaze path variance was explained by decoding up to three positions per TR than by decoding only one position per TR (Fig. 2f). In this dataset, which featured a TR of 1 s (ref. ^15^), this corresponds to a decoding resolution of 3 Hz, which dovetails with the average visual search eye movement frequency of 3 Hz (ref. ^17^). Moreover, the ten real and decoded sub-TR labels varied similarly within each TR (Extended Data Fig. 8b) and across TRs (Extended Data Fig. 8c,d), which again suggests that within-TR movements could be detected. While the exact resolution likely depends on the viewing behavior and the imaging protocol, these results show that at least a moderate subimaging temporal decoding resolution is indeed feasible.

### Across-dataset generalization

The results presented so far show that the gaze decoding with DeepMReye is highly accurate when the viewing behavior and the imaging protocol are similar between training and test sets. To test if our model also generalizes across datasets, we next implemented a leave-one-dataset-out cross-validation scheme. Most datasets were acquired by different groups using different MR scanners, participants and viewing behaviors but with similar voxel sizes and TRs. While this across-dataset scheme led to overall lower performance scores than the across-participant (within-dataset) scheme presented earlier, it nevertheless recovered viewing behavior with remarkable accuracy in all cases (median performance of the 80% most reliable participants (low PE): all datasets, *r* = 0.84, *R*^2^ = 0.59, EE = 2.78°, FoS = 13.8%; fixation, *r* = 0.79, *R*^2^ = 0.52, EE = 5.34°, FoS = 22%; pursuit 1, *r* = 0.88, *R*^2^ = 0.64, EE = 1.47°, FoS = 14%; pursuit 2, *r* = 0.86, *R*^2^ = 0.65, EE = 2.15°, FoS = 12%; pursuit 3, *r* = 0.85, *R*^2^ = 0.55, EE = 2.01°, FoS = 9%; free viewing, *r* = 0.84, *R*^2^ = 0.61, EE = 2.96°, FoS = 12%; Extended Data Fig. 3). This suggests that datasets acquired with similar fMRI protocols can be used for model training, even if the recording site or the protocol were not exactly the same. Future investigations will need to quantify how larger differences in scan parameters affect this across-dataset generalization (for example, different phase-encoding directions or slice tilts). The across-dataset generalization is expected to improve in the future as more datasets are being used for model training.

### Robust across voxel sizes and TRs

**Fig. 3:**
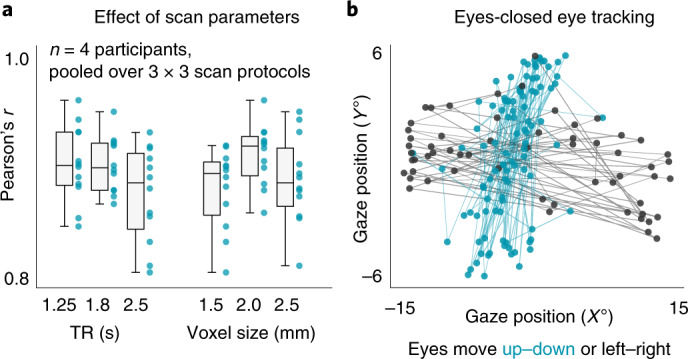
Effect of scan parameters and eye tracking while the eyes are closed. **a**, Effect of voxel size and TR. Gaze decoding expressed as the Pearson correlation between true and decoded gaze trajectory for different voxel sizes and TRs is plotted. Shown are whisker box plots (central line, median; box, 25th and 75th percentile; whisker, all data points not considered outliers; outliers, data points outside 1.5× interquartile range) and single-participant data (*n* = 4) for nine fMRI protocols collapsed either over TR or voxel size. DeepMReye recovered viewing behavior successfully in all sequences tested. **b**, Decoded gaze coordinates for a participant being instructed to move the eyes left and right or up and down while keeping them closed. Dots are colored based on button press of the participant, indicating movement direction. Source data are provided. Source data

fMRI protocols can differ in many aspects. Most importantly, in this context, they can differ in the spatial and temporal resolution of the acquired data (that is, voxel size and TR). To explore the influence of these two parameters on the decoding performance in detail, we varied them systematically across nine fMRI protocols for the acquisition of a sixth dataset. For each of the nine sequences, we scanned four participants with concurrent camera-based eye tracking while they freely explored pictures^18^ or performed fixation^11^ and smooth pursuit tasks similar to the ones used earlier^12–14^. DeepMReye decoded gaze position robustly in this dataset 6 during all of these tasks and in all imaging protocols tested (3 × 3 design: TR = 1.25 s, 1.8 s, 2.5 s; voxel size = 1.5 mm, 2 mm, 2.5 mm; Fig. 3a), demonstrating that it is widely applicable across a broad range of routinely used voxel sizes and TRs.

### Eyes-closed tracking

Traditional MR-compatible eye-tracking systems typically detect certain features of the eyes, such as the pupil and/or the corneal reflection in a video, which are then tracked over time^19^. When the relevant features are occluded or cut off on the video (for example, when the eyes close), the tracking is lost. Because our approach relies on the fact that the eyeball MR signal changes as a function of gaze position (Fig. 1b), it might be possible to decode gaze position, or in this case more generally the state of the eyeballs, even when the eyes are closed. As a proof of concept, we therefore tested in one participant of dataset 6 whether DeepMReye can uncover viewing behavior even when the eyes are closed. The participant was instructed to close the eyes and move them either repeatedly from left to right or from top to bottom and to indicate the behavior via a key press. We trained DeepMReye on the diverse eyes-open viewing data from all participants in dataset 6 and then decoded from the one participant while the eyes were closed. We found that the gaze pattern decoded with DeepMReye closely matched the participant’s self-report (Fig. 3b), suggesting that it is indeed possible to perform eye tracking while the eyes are closed.

### Eyes-open versus eyes-closed classification

**Extended Data Fig. 9:**
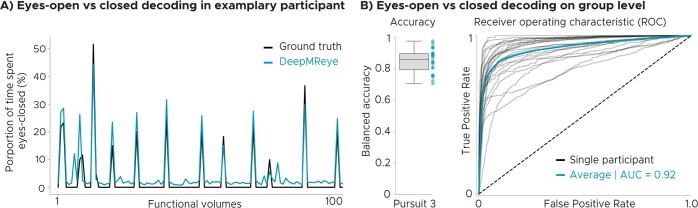
Eyes-open vs. eyes-closed across-participant decoding in smooth pursuit dataset 3. A) Single-participant example of decoding the proportion of time spent eyes-closed. Note that model accuracy and hit rates were computed on binarized decoding labels, but that the model output is the actual proportion of time spent eyes-closed as shown on in this panel. B) Group-level accuracy for decoding whether the eyes were open or closed for more than 10% of the time it took to acquire the respective functional volume (left panel). We plot whisker-box-plots (central line: median, box: 25th and 75th percentile, whisker: all data points not considered outliers, outliers: data points outside 1.5x interquartile range) and single-participant data (dots). We calculated balanced accuracy to rule out that the results reflect the model always classifying the most common label. In addition, we plot a Receiver operating characteristic (ROC) curve of the group-level data (right panel). Source data

When the eyes are closed, the eyelid pushes down onto the eyeball, which changes its shape slightly^20^. Because this should affect the MRI signal obtained from the eyes, we therefore tested if our model could also decode if the eyes were open or closed. To do so, we used the camera-based eye-tracking labels of our smooth pursuit dataset 4 (ref. ^14^) to compute the proportion of time spent eyes closed for each volume of each participant. These data were then used to train and test a variant of our across-participant decoding model using our 80%/20% data cross-validation procedure (Methods). We found that we could indeed recover the proportion of time spent eyes closed with high reliability from each volume (Extended Data Fig. 9a), which we then combined to obtain a continuous decoded time series. This time series then served as the basis for classification. To binarize it, we thresholded it at various thresholds (for example, at 10%; Extended Data Fig. 9b) and then computed hit rates and accuracy of our model. This showed that DeepMReye could indeed reliably predict whether the eyes were open or closed for various proportions of the TR (for example, accuracy for 10% cutoff is 89.9% ± 0.05, balanced accuracy is 84.6% ± 0.07 and area under the curve is 0.92), showing that the eyes-open versus eyes-closed classification is indeed feasible. Note that the model output is the continuous, unthresholded time series (Extended Data Fig. 9a), and users may implement different classification procedures or regression analyses according to their needs.

### Viewing behavior explains network-wide brain activity

The results presented so far demonstrate that DeepMReye can be used to perform eye tracking in many experimental settings. A critical open question that remained was whether its decoding output can be used to analyze brain activity. To test this, we implemented a whole-brain mass-univariate general linear model (GLM) for the visual search dataset 5. We again chose this dataset because it featured the most complex viewing pattern tested. To simulate differences in viewing behavior between the two conditions, we first computed an eye movement index, reflecting the Euclidean distance between gaze positions of subsequent volumes. We used this eye movement index to build two main regressors of interest, one modeling large eye movements and one modeling short eye movements. Both regressors were binarized and convolved with the hemodynamic response function. Contrasting the model weights estimated for these two regressors was expected to reveal regions in the brain whose activity is driven by viewing behavior, such as the visual and oculomotor (attention) network^3,4^.

To know what we were looking for, we first conducted this analysis using the gaze labels obtained with traditional camera-based eye tracking and then compared the results to the ones obtained for the three cross-validation schemes of DeepMReye (within participants, across participants and across datasets).

**Fig. 4:**
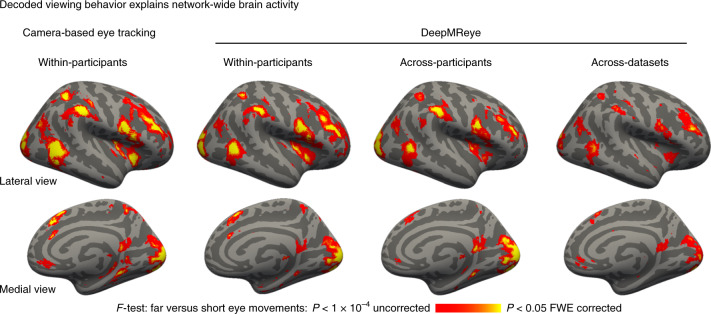
Decoded viewing behavior explains network-wide brain activity. GLM group results for the contrast ‘far versus short eye movements’ during visual search. We plot the *F*-statistic of this contrast superimposed on a template surface (fsaverage) for gaze labels obtained with camera-based eye tracking (left) as well as for three DeepMReye cross-validation schemes. For the within-participants scheme, all participants of a dataset were included with different partitions in model training and testing. For the across-participants scheme, different participants were included during model training and testing. For the across-datasets scheme, different datasets (and hence also different participants) were included during model training and testing; FWE, family-wise error. Source data are provided. Source data

As predicted, we found that viewing behavior explained brain activity in a large network of regions (Fig. 4), including the early visual cortex, frontoparietal regions (likely the frontal eye fields), the posterior parietal cortex as well as temporal lobe regions (likely including the human motion complex)^21^. Importantly, however, differences in viewing behavior also explained brain activity in regions not typically associated with oculomotor function, such as the ventromedial prefrontal cortex, the anterior and posterior cingulate cortex, the medial parietal lobe (likely comprising the retrosplenial cortex), the parahippocampal gyrus and the hippocampus (Fig. 4).

Strikingly, comparing the results obtained with DeepMReye to the ones obtained with camera-based eye tracking showed an exceptional match between the two (Fig. 4). This was true even for the across-participant decoding scheme, which can be conducted even in existing datasets with some preparation (Fig. 2; see online user documentation). Moreover, even the across-dataset scheme explained gaze-related variance on the group level, despite the differences in the underlying viewing behaviors and imaging protocols.

**Extended Data Fig. 10:**
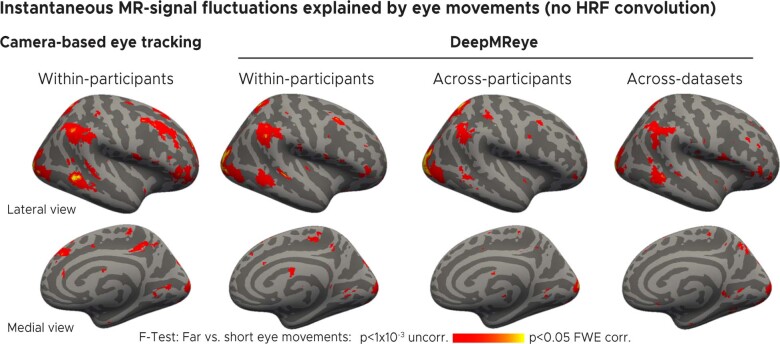
General-linear-model (GLM) group results for the contrast ‘Far vs. short eye movements’ during visual search without accounting for the hemodynamic response function. We plot the F-statistic of this contrast superimposed on a template surface (fsaverage) for gaze-labels obtained with camera-based eye tracking (first panel) as well as for three DeepMReye cross-validation schemes. Within-participants: All participants of a dataset were included with different partitions in model training and test. Across-participants: Different participants were included during model training and test. Across-datasets: Different datasets (and hence also different participants) were included during model training and test. Source data

Finally, because eye movements are associated not only with brain activity but also with imaging artifacts, the MRI signal might also be affected instantaneously when the movement occurs. To quantify these instantaneous effects, we repeated the GLM analysis modeling eye movement-related fluctuations in the MRI signal without accounting for the hemodynamic response. Again, we found that eye movements explained signal variations in many brain regions (Extended Data Fig. 10), likely reflecting a combination of imaging artifacts and instantaneous hemodynamic components (for example, the initial dip). Together, these analyses demonstrate that eye movements explain a large amount of MR signal variance throughout the brain even when accounted for at image-wise resolution, which is similar to, but independent from, head motion. This variance is not captured by traditional head motion regressors (Supplementary Fig. 4).

## Discussion

DeepMReye is a cameraless eye-tracking framework based on a CNN that decodes gaze position from the MR signal of the eyeballs. It allows monitoring of viewing behavior accurately and continuously at a moderate subimaging resolution without the need for MR-compatible cameras. We demonstrated that our approach works robustly for a wide range of voxel sizes and repetition times as well as for various viewing behaviors, including fixation, smooth pursuit, free viewing and, as a proof of concept, even when the eyes were closed. For each gaze position and participant, the model outputs an unsupervised PE score that can be used to filter out outliers even when test labels are missing. A small training set can yield a well-trained model and high decoding performance even when trained without camera-based labels. Using an easy-to-implement analysis, we showed that the decoded gaze positions and eye movements can be used in subsequent fMRI analyses similar to camera-based eye tracking, and doing so revealed gaze-related activity in a large network of regions in the brain^3,4,6,21^. Critically, by testing our model in independent participants within each dataset, but also across datasets acquired with different MR scanners and protocols, we demonstrated the potential of DeepMReye to successfully decode viewing behavior also in existing fMRI data.

### MR-based gaze prediction

The present work is directly inspired by earlier reports showing that the MR signal of the eyeballs can be used to infer the state of the eyes during MRI scanning. This includes movements of the eyes^7,8,22,23^, the position of gaze on the screen^9,10,22,24,25^ or whether the eyes were open or closed^20^. Moreover, gaze position can be decoded from early visual cortex activity during scene viewing^26^ and, as shown here, during visual search (Fig. 2e). However, DeepMReye goes beyond these earlier reports in multiple ways. Most importantly, earlier approaches, such as predictive eye estimation regression^10^, required calibration data for every single participant, meaning that at least two calibration scans need to be acquired during each scanning session. By contrast, our deep learning-based approach generalizes across participants, allowing for eye tracking even when training and test labels are missing. The model could be trained on the data of a few participants and then used for decoding from all other participants. Moreover, earlier approaches were limited to the sampling resolution of the imaging protocol, meaning that one average gaze position per functional image could be extracted. By contrast, we extracted gaze position at a moderate sub-TR resolution (~3 Hz) and with higher accuracy than previous approaches, allowing us to perform MR-based eye tracking with a higher level of detail. As a proof of principle, we show that our model reconstructs viewing behavior even when the eyes are closed. Finally, we provide an open source and user-friendly implementation for MR-based eye tracking as an interactive pipeline inspired by other fMRI open source initiatives (for example, see ref. ^27^). Hence, DeepMReye overcomes several critical limitations of earlier work, presenting the most general and versatile solution to cameraless eye tracking in MRI to date.

### What information does the model use?

Eye movements naturally entail movements of the eyeballs but also of the optic nerves and the fatty tissue around them. To capture these movements, our custom eye masks cover a large area behind the eyes excluding skull and brain tissue. When the eyes move, the multivoxel pattern in these masks changes drastically (Fig. 1b), an effect that might even be amplified by the magnetic field distortions often occurring around the eyes. Hence, DeepMReye likely uses information traditionally considered to be motion artifacts, which are not corrected by classical realignment during preprocessing (Supplementary Fig. 4 and Extended Data Fig. 10). The fact that the actual orientation and motion of the eye are used for decoding (Supplementary Fig. 2) also means that no hemodynamic lag needs to be considered (Fig. 2e) and that both conjugate and disconjugate (vergence) eye movements could be detected. The current gaze position is decoded directly from each respective TR. Moreover, we believe that two sources of information further contribute to the moderate subimaging decoding resolution that we observed. First, different imaging slices are being acquired at a different time within each TR and thus inherently carry some sub-TR information. This is true also for fMRI protocols that use multiband acquisition, which includes all datasets tested here. Future studies could examine the effect of slice timing on decoding resolution in more detail. Second, similar to motion blur in a long-exposure camera picture, the MRI signal intensity of a voxel can itself be affected by movements. The multivoxel pattern at each TR might therefore reflect how much the eyes moved, and the same average gaze position might look different depending on which positions were sampled overall within the respective TR. An exciting avenue to be explored in future studies is the influence of scanning sequence parameters, such as slice timing on the decoding, which may potentially reveal certain settings that further improve the temporal resolution of our model.

### Looking forward

DeepMReye offers a multitude of exciting applications ranging from simple behavioral monitoring over confound removal to new and improved task-based analyses. Most basically, it offers an additional and low-effort behavioral readout for any fMRI experiment and allows for monitoring task compliance, for example, by verifying that a fixation cross was fixated. Removing samples at which fixation was not maintained from subsequent analysis has been shown to improve predictive modeling results^24^ and may help to reduce the effects of in-scanner sleep more easily^28^.

Our approach enables studies of the relationship between viewing and brain activity and may more generally be used to inform almost any type of task-based model about the underlying viewing behavior. This could, for example, further improve the explanatory power of predictive models^29,30^ and be especially promising for naturalistic free-viewing paradigms because the currently attended aspect of a stimulus can be taken into account^31^. This may prove especially fruitful in research domains that do not typically use eye tracking to date^6,32,33^.

Importantly, eye movements can also be a major source of confounds in neuroimaging studies. As mentioned earlier, if differences in viewing between two conditions remain undetected, the interpretation of neuroimaging results may be compromised. We demonstrated here that many brain regions are affected by this issue, many of which are not typically studied in the context of eye movements (Fig. 4). Moreover, eye movements are associated with imaging artifacts that can affect data integrity throughout the brain^5^. A popular way of minimizing such confounds is having participants fixate at a fixation cross, which is helpful but also puts artificial constraints on a behavior that is fundamental to how we explore the world. Moreover, task compliance cannot always be guaranteed. DeepMReye may reduce confounds and artifacts, for example, by adding eye movement regressors directly to a GLM analysis as it is standard practice for head motion regressors. Additionally, it could be used to track if the eyes were open during scanning, potentially helping to identify the effects of fatigue and frequent blinking on fMRI activity. This promises to improve the interpretability of task-based and resting-state fMRI results alike because nuisance variance would no longer be assigned to the regressors of interest^34^.

DeepMReye can thus provide many experimental and analytical benefits that traditional eye tracking systems can provide. Critically, it does so without requiring any expensive equipment, trained staff or experimental time. It can be used widely in both research and clinical settings, for example, to study or diagnose neurodegenerative disorders^1^. Excitingly, it can even go beyond traditional eye tracking in certain aspects, offering new experimental possibilities that cannot easily be realized with a camera. For example, we showed as a proof of principle in one participant that eye movements can be tracked even while the eyes are closed. This suggests that our approach could be used to study oculomotor systems in the total absence of visual confounds, during resting state^33^ and potentially even during REM sleep. While future studies will need to validate the accuracy of the eyes-closed decoding in a larger sample, it promises many new research questions to be addressed. Moreover, the across-participant generalization enables new studies of participant groups for which camera-based eye trackers are not applicable. For example, DeepMReye could be trained on the data of healthy volunteers and then tested on blind participants for whom camera-based eye trackers cannot be calibrated. Most importantly, it allows gaze decoding in many existing fMRI datasets, and it could hence make new use of large and instantly available data resources.

Finally, the same model architecture can be used to decode gaze position from brain activity directly as well. Doing so is as simple as replacing the eye masks by a regions-of-interest mask of a certain brain region and accounting for the hemodynamic lag. We demonstrated this possibility using fMRI data from area V1 (Fig. 2e). Likewise, the same decoding pipeline could be used to decode other behavioral or stimulus features from brain activity or even to classify diseases based on MRI images, again showing the power of deep learning-based methods for image analysis and neuroscience in general^35,36^.

### Limitations and further considerations

It is important to note that DeepMReye also has certain limitations and disadvantages compared to camera-based eye tracking. First, the eyeballs need to be included in the MRI images. This may not always be possible and can affect the artifacts that eye movements can induce. In practice, however, many existing and future datasets do include the eyes, and even if not, DeepMReye could still be used to decode from brain activity directly. Second, the PE allows for detection of outlier participants accurately, but it should not be regarded as an absolute measure of decoding quality. We recommend users to further consider other measures to assess the reliability of the model and data quality (see online user documentation). Our pipeline generates interactive quality check visualizations to support this process (Supplementary Fig. 3). Third, despite decoding at a temporal resolution that is higher than the resolution of the imaging protocol, our approach does by no means reach the temporal resolution of a camera. Many aspects of viewing behavior happen on a time scale that can hence not be studied with DeepMReye. For experiments requiring such high temporal resolution, for example, for studying individual saccades and associated variables such as saccade latency or amplitude, we therefore recommend a camera system.

However, we believe that many fMRI studies could benefit immensely from eye tracking even at a moderate temporal resolution, and many research questions do not require high resolution to be addressed. For example, we showed that eye movements explain activity in many brain regions even when modeled at image-wise resolution (Fig. 4), which is similar to, but independent from, head motion. While we encourage users to implement their own analyses based on the decoding output, these and other results show that even simple regression analyses of image-wise eye movements can provide key insights into human brain function and clean up noise^10,14,15,24,26^. Moreover, many regression analyses in neuroimaging require the eye-tracking data to be downsampled to the imaging resolution irrespective of the acquisition sampling rate. This means that even if gaze behavior was monitored at 1,000 Hz with a camera, the effective eye-tracking data resolution entering fMRI analysis is often the same as the one of DeepMReye.

Most importantly, however, a large number of researchers, MRI facilities and hospitals do not have access to MR-compatible cameras, leaving MR-based eye tracking as the only available option. The decision is thus most often to be made not between MR-based and camera-based eye tracking but between MR-based eye tracking and no eye tracking at all. To date, the latter option is the most common scenario, which we see as a missed opportunity. DeepMReye allows monitoring gaze without a camera with a moderate temporal resolution even in many existing datasets and when the eyes are closed. It therefore complements camera systems for situations in which these are not applicable, and it provides an open source alternative to cameras for research that would otherwise be conducted without eye tracking.

## Conclusions

In sum, DeepMReye is a cameraless deep learning-based eye tracking framework for fMRI experiments. It works robustly across a broad range of gaze behaviors and imaging protocols, allowing for the reconstruction of viewing behavior with high precision even in existing datasets. This work emphasizes the importance and the potential of combining eye tracking, neuroimaging and artificial intelligence for studying human brain function, and it provides a user-friendly and open source software that is widely applicable post hoc.

## Methods

### Datasets

DeepMReye was trained and tested on data from 268 participants acquired on five 3T MRI scanners with 14 different scanning protocols and various preprocessing settings. The individual datasets are described below and were partially used in earlier reports. For other details of each individual dataset, please see the original published articles^11–15^. A T1-weighted structural scan with 1-mm isotropic voxel resolution was acquired for all participants, and camera-based eye-tracking data were included for participants in datasets 3–6. An overview of the datasets is provided in Supplementary Table 1.

#### Dataset 1: fixation and saccades

Data and task These data were made publicly available by Alexander and colleagues^11^ and were downloaded from the Healthy Brain Network (HBN) Biobank (http://fcon_1000.projects.nitrc.org). These data were also used in earlier reports^10^. It is part of a larger and ongoing data collection effort with a pediatric focus and comprises participants between 5 and 21 years of age. We limited our analysis to a subset of the full dataset for which we ensured that there were no visible motion artifacts in either the T1- or the average T2-weighted images, and the eyeballs were fully included in the functional images. We included 170 participants in total. For each participant, at least two fMRI runs were scanned in which they performed a typical eye-tracking calibration protocol. In each run, they fixated at a fixation target that sequentially moved through 27 locations on the screen, with each location being sampled twice for 4 s. Gaze positions were sampled within a window of *X* = 19° and *Y* = 15° visual angle. The screen coordinates of the fixation target served as training and testing labels for the main analyses (Fig. 2).

Note that this dataset included children and thus likely relatively inexperienced participants who did not always fixate accurately, and it featured the least amount of single-participant data of all datasets tested. This may have contributed to a larger variance in model performance scores on group level than other datasets (Fig. 2c). Moreover, it featured the largest data range and thus the largest possible outliers, which may explain why the within-participant outlier removal had a stronger positive effect on model performance in this dataset than the others (Extended Data Fig. 4).

fMRI data acquisition and preprocessing Imaging data were acquired on a Siemens 3T Tim Trio MRI scanner located at the Rutgers University Brain Imaging Center, Newark, New Jersey, USA. The following echo-planar imaging (EPI) parameters were used: voxel size = 2.4 mm isotropic, TR = 800 ms, TE = 30 ms, flip angle = 31° and multiband factor = 6. Images were co-registered to our template space as described below.

#### Dataset 2: smooth pursuit 1

Data and task These data were used in one of our previous reports^12^. Nine participants performed a smooth pursuit visual tracking task in which they either tracked a fixation target moving on a circular trajectory with a radius of 8° visual angle or one that remained at the screen center. In addition, planar dot motion stimuli were displayed in the background moving on the same circular trajectory at various speeds. This resulted in a total of nine different conditions. The following pursuit and motion speed combinations were tested in separate trials: eye, background in degrees per s = (0, 0), (0, 1), (0, 3), (2, 1), (2, 2), (2, 3), (3, 2), (3, 3), (3, 4). These conditions were tested in blocks of 12 s in the course of 34 trials over four scanning runs of ~10.5 min each. To balance attention across conditions, participants performed a letter repetition detection task displayed on the fixation target. Gaze positions were sampled within a window of *X* = 8° and *Y* = 8° visual angle. The screen coordinates of the fixation target served as training and testing labels for our model.

fMRI data acquisition and preprocessing Imaging data were acquired on a Siemens 3T MAGNETOM Prisma MRI scanner located at the Max-Planck-Institute for Biological Cybernetics in Tubingen, Germany. The following EPI parameters were used: voxel size = 2 mm isotropic, TR = 870 ms, TE = 30 ms, flip angle = 56°, multiband factor = 4 and generalized autocalibrating partial parallel acquisition (GRAPPA) factor = 2. Note that nine other participants were excluded because the functional images did not or only partially included the eyeballs. Images were corrected for head motion and field distortions using SPM12 (www.fil.ion.ucl.ac.uk/spm/) and then co-registered to our template space as described below.

Eye tracking We monitored gaze position at 60 Hz using a camera-based eye tracker by Arrington Research. Please note that these eye-tracking data showed a higher noise level than the other datasets due to drift and because the pupil was frequently lost. We therefore used the screen coordinates of the fixation target for model training and testing as in dataset 1. To still visually compare the decoding output to the eye-tracking data post hoc, we removed blinks, detrended the eye-tracking time series using a second-order polynomial function and median centered it on the screen center. We removed samples in which the pupil was lost by limiting the time series to the central 14° × 14° visual angle, smoothed it using a running average kernel of 100 ms and scaled it to match the data range of the fixation target using the sum of squared errors as loss function. The time series was then split into the individual scanning acquisitions.

#### Dataset 3: smooth pursuit 2

Data and task These data were used in one of our previous reports and comprised 34 participants^13^. Like in dataset 2, participants performed a smooth pursuit visual tracking task in which they fixated at a fixation target moving on a star-shaped trajectory. Twenty-four eye movement directions were sampled in steps of 15° at four speed levels, 4.2° per s, 5.8° per s, 7.5° per s and 9.1° per s. Speeds were interleaved and sampled in a counterbalanced fashion. In addition to the visual tracking task, participants performed a time-to-collision task. The trajectory was surrounded by a circular yellow line on a gray background with a radius of 10° visual angle centered on the screen center. Whenever the fixation target stopped moving before switching direction, participants indicated by button press when the target would have touched the yellow line if it continued moving. Gaze positions were sampled within a window of *X* = 10° and *Y* = 10° visual angle. Each participant performed a total of 768 trials in the course of four scanning runs with 16–18 min (including a short break in the middle). The screen coordinates of the fixation target served as training and testing labels for the main analyses (Fig. 2).

fMRI data acquisition and preprocessing Imaging data were acquired on a Siemens 3T MAGNETOM Skyra located at the St. Olavs Hospital in Trondheim, Norway. The following EPI parameters were used: voxel size = 2 mm isotropic, TR = 1,020 ms, TE = 34.6 ms, flip angle = 55° and multiband factor = 6. Images were corrected for head motion using SPM12. The FSL topup function was used to correct field distortions using an image acquired with the same protocol except that the phase-encoding direction was inverted (https://fsl.fmrib.ox.ac.uk/fsl/fslwiki/topup). Images were then co-registered to our template space as described below.

Eye tracking We monitored gaze position during the experiment at a rate of 1,000 Hz using an MR-compatible infrared-based eye tracker (Eyelink 1000). Blinks were removed, and the time series was downsampled to 100 Hz, linearly detrended within each scanning run and smoothed with a running average kernel of 100 ms. We then split the time series into individual scanning acquisitions (TRs) to obtain the final training and testing gaze labels for our model. The camera-based eye-tracking labels served as training and testing labels for supplementary analyses (Extended Data Fig. 5).

### Dataset 4: smooth pursuit 3

Data and task These data were used in one of our previous reports^14^. Twenty-four participants performed a smooth pursuit visual tracking task in which they tracked a fixation target moving at a speed of 7.5° per s on a star-shaped trajectory with 36 directions. The target moved within a virtual arena, which participants oversaw from a bird’s-eye view. Eye movement directions were sampled in steps of 10°. In a visual motion control condition, the target remained at the screen center, and the arena moved instead. Participants additionally performed a spatial memory task. They memorized the location of colored objects on the screen, which were shown only when the fixation target moved across them. Gaze positions were sampled within a window of *X* = 15° and *Y* = 15° visual angle. Each participant performed a total of 81 trials in the course of nine scanning runs. This included 54 smooth pursuit trials of 60 s each and 27 center fixation trials of 30 s each. The screen coordinates of the fixation target served as training and testing labels for the main analyses (Fig. 2).

fMRI data acquisition and preprocessing Imaging data were acquired on a Siemens 3T MAGNETOM PrismaFit MRI scanner located at the Donders Centre for Cognitive Neuroimaging in Nijmegen, the Netherlands. The following EPI parameters were used: voxel size = 2 mm isotropic, TR = 1,000 ms, TE = 34 ms, flip angle = 60° and multiband factor = 6. Data were realigned using SPM12 (https://www.fil.ion.ucl.ac.uk/spm/) and co-registered to our template space as described below.

Eye tracking Similar to dataset 3, we again monitored gaze position during the experiment at 1,000 Hz using an Eyelink 1000 eye tracker. Blinks were removed, and the data were downsampled to the monitor refresh rate of 60 Hz. We then reduced additional tracking noise by removing samples at which the pupil size diverged more than one standard deviation from the mean by removing the intertrial interval during which most blinks occurred and by smoothing the time series with a running average kernel of 100 ms. We then linearly detrended and median centered the time series of each trial individually to remove drift. Finally, we split the time series according to the underlying scanner acquisition times to create our final training and testing labels for this dataset. Note that the original dataset^14^ includes five additional participants for which no eye-tracking data have been obtained and were excluded. The camera-based eye-tracking labels served as training and testing labels for supplementary analyses (Extended Data Fig. 5).

#### Dataset 5: visual search

Data and task These data were kindly provided by Julian and colleagues^15^. Twenty-seven participants performed a self-paced visual search task, searching for the letter ‘L’ in a search display filled with distractor letter ‘T’. Following detection, participants pressed a button. Each trial lasted for an average of 7.50 s, followed by fixation at the screen center for 2–6 s. The number of distractors varied over trials between 81, 100, 144, 169 or 121. Participants performed either four or six runs of 6.5 min each. Task-relevant gaze positions were sampled within a window of *X* = 17° and *Y* = 17° visual angle. Camera-based eye-tracking data were acquired and served as training and testing labels for our model (see below).

fMRI data acquisition and preprocessing Imaging data were acquired on a Siemens 3T MAGNETOM Prisma MRI scanner located at the Center for Functional Imaging in Philadelphia, Pennsylvania, USA. The following EPI parameters were used: voxel size = 2 mm isotropic, TR = 1,000 ms, TE = 25 ms, flip angle = 45° and multiband factor = 4. Images were corrected for head motion using SPM12 and co-registered to our template space as described below. Note that the original dataset includes nine more participants whose eyeballs were cut off on the functional images and were excluded here.

Eye tracking Gaze position was monitored at 30 Hz using the camera-based eye tracker LiveTrack AV by Cambridge Research Systems. We median centered the time series and removed tracking noise by limiting the time series to values within the central 40 × 40 visual degree. We then split the data into individual scanning acquisitions to obtain the final gaze labels for model training and testing.

#### Dataset 6: fixation, smooth pursuit, free viewing and eyes-closed eye movements

Data and task Four male participants performed four viewing tasks while imaging data were acquired in the course of nine scanning runs using nine EPI protocols (one per run) along with concurrent camera-based eye tracking. First, they fixated sequentially at 37 locations on the screen for 2 s each starting in the screen center. The locations were determined using a custom random-walk algorithm that balanced the sampling of 12 directions (30° steps) and distances between the fixation points (4°, 8° or 12° visual angle). Next, they performed a smooth pursuit version of this random-walk task for which we linearly interpolated the trajectory between fixation points. This resulted in a target moving sequentially into 12 directions at a speed of 2° per s, 4° per s or 6° per s, changing to a randomly selected direction and speed every 2 s. Next, participants freely explored 30 sequentially presented images of everyday objects for 3 s each. The images were randomly drawn from the THINGS database^18^. Finally, participants closed their eyes and moved them either from left to right or from top to bottom for a total of 105 s. Switches between horizontal and vertical movements were indicated via button press.

fMRI data acquisition and preprocessing Imaging data were acquired using nine EPI sequences on a Siemens 3T MAGNETOM Skyra located at St. Olavs Hospital in Trondheim, Norway. The sequences featured three repetition times and three voxel sizes in a 3 × 3 design. All images were corrected for head motion using SPM12 and co-registered to our template space as described below. See Supplementary Table 2 for parameter details. Data acquisition was approved by the regional committees for medical and health research ethics (REK sør-øst), Norway, and participants gave written informed consent before scanning.

Eye tracking Gaze position was monitored during the experiment at 1,000 Hz using an Eyelink 1000 eye tracker. Tracking noise was reduced by excluding samples at which the pupil size diverged more than two standard deviations from the mean. Blinks were removed. The time series was downsampled to 60 Hz and median centered based on the median gaze position of the free viewing condition within each scanning run. We then split the time series into individual scanning acquisitions to obtain the final training and testing gaze labels for our model.

### Eye masks, co-registration and normalization

Eye masks were created by manually segmenting the eyeballs including the adjacent optic nerve, fatty tissue and muscle area in the Colin27 structural MNI template using itkSNAP (http://www.itksnap.org; Fig. 1a). We then created a group average functional template by averaging the co-registered functional images of 29 participants. These were acquired while the participants fixated at the screen center for around 13 min each in the course of a longer scanning session^14^. To ensure that the final eye masks contain the eyeballs of every participant, all imaging data underwent three co-registration steps conducted using Advanced Normalization Tools (ANTs) within Python (ANTsPy). First, we co-registered each participant’s mean EPI non-linearly to our group-level average template. Second, we co-registered all voxels within a bounding box that included the eyes to a preselected bounding box in our group template to further improve the fit. Third, we co-registered the eyeballs to the ones of the template specifically. Importantly, all data in our group average template reflected gaze coordinates (0, 0), which is the screen center. This third eyeball co-registration hence centered the average gaze position of each participant on the screen. We did this to improve the fit but also because it aligned the orientation of the eyeballs relative to the screen across participants. Finally, each voxel underwent two normalization steps. First, we subtracted the across-run median signal intensity from each voxel and sample and divided it by the median absolute deviation over time (temporal normalization). Second, for each sample, we subtracted the mean across all voxels within the eye masks and divided by the standard deviation across voxels (spatial normalization). The fully co-registered and normalized voxels inside the eye masks served as model input.

Note that the average gaze centering procedure is fully optional. Without it, however, two participants fixating the same screen location may gaze into different directions depending on the mirror placement inside the MRI. Our model assumption overcomes this problem, is valid for all datasets tested here (Supplementary Fig. 1) and will likely be valid in many other datasets as well. However, users may choose not to use it, or they could estimate potential offsets to the screen center in their own training data and factor it in after obtaining the decoded gaze coordinates. DeepMReye can thus also be used for datasets in which the average gaze position does not reflect the screen center.

### Model architecture

DeepMReye is a CNN that uses 3D data to classify a 2D output, the horizontal (*X*) and vertical (*Y*) gaze coordinates on the screen. The model uses the voxel intensities from the eye masks as input and passes it through a series of 3D convolutional layers interleaved with group normalization and non-linear activation functions (mish^37^). In detail, the eye mask (input layer) is connected to a 3D convolutional block with a kernel size of 3 and strides of 1, followed by dropout and a 3D convolutional downsampling block, which consists of one 3D convolution followed by a 2 × 2 × 2 average pooling layer. After this layer, we use a total of six residuals blocks, in which the residual connection consists of one 3D convolutional block concatenated via simple addition. Each residual block consists of group normalization, non-linear activation and a 3D convolution, which is applied twice before being added to the residual connection. This results in a bottleneck layer consisting of 7,680 units, which we resample to achieve sub-TR resolution (see details below). The time resolution dictates the number of resampled bottleneck layers, with, for example, ten resampled layers producing a 10 times higher virtual resolution than the original TR.

The bottleneck layer carries an abstracted low-dimensional latent representation of the input, which is used to train two fully connected layers for decoding. The first dense layer learns to decode gaze position by minimizing the EE between the predicted gaze position and the ground truth gaze position as a loss function. The second dense layer in turn predicts the EE used to train the first dense layer. This allowed us to obtain an unsupervised EE for each decoded gaze sample even when test labels were missing. We refer to this predicted, unsupervised EE as the PE. It indicates how certain the model is about its own gaze decoding output and is strongly correlated with the real EE in our test data (Fig. 2b and Extended Data Fig. 1). The second dense layer basically learns how the input normally looks when the error is low versus when it is high (for example, when the data are not well aligned or the eyeballs are missing; Supplementary Fig. 3). If the unsupervised error is high, the model itself anticipates that the decoded gaze position likely diverges much from the real gaze position. Accordingly, samples with high PE should not be trusted. DeepMReye is trained using a combination of the two losses, the EE (90% weighting) and the PE loss (10% weighting) as described in detail below.

### Model optimization and training

Hyperparameters were optimized using random search, which we monitored using the ‘Weights & Biases’ model tracking tool^38^. The following parameters were optimized: the learning rate (0.001–0.00001), the number of residual blocks (depth, 3–6), the size of the filters (16–64), the filter multiplier per layer (1–2; for example, 32, 64, 128 uses a multiplier of 2), the activation function (relu, elu, mish), the number of groups in the group normalization (4, 8, 16), the number of fully connected layers (1, 2), the number of units in each fully connected layer (128–1,024) and the dropout rate (0–0.5). In addition, to further improve the generalizability of our model, we added the following data augmentations to the model training: input scaling, translations (*X*, *Y*, *Z*) and rotations (azimuth, pitch and roll), which were applied on each sample.

The model was trained using a variant of stochastic gradient descent (Adam) as a learning algorithm^39^ and a batch size of 8 to train the model. Because considering samples from different participants improved model performance in an earlier version of our pipeline, we mixed samples in each training batch to represent 3D inputs from different participants. For estimating the loss between real and predicted gaze position, we used the EE

1 $${{{{\mathcal{L}}}}}_{\rm{ED}}=\sqrt{\mathop{\sum }\limits_{i=1}^{M}{({\hat{y}}_{i}-{y}_{i})}^{2}}$$

with *y*_*i*_ as the real gaze position and ${\hat{y}}_{i}$ as the predicted gaze position. For calculating the PE, which reflects an unsupervised estimate of the EE, we used the mean squared error between real and predicted EE, which itself has been computed using the real and predicted gaze path as described above. The PE was computed as

2 $${{{{\mathcal{L}}}}}_{\rm{MSE}}={\frac{1}{M}}{\mathop{\sum }\limits_{i=1}^{M}}{\left({\hat{y}}_{i}-{{y}_{i}}\right)}^{2}$$

with *y*_*i*_ being the EE between real and predicted gaze path (${{{{\mathcal{L}}}}}_{ED}$) and ${\hat{y}}_{i}$ being the predicted EE for this sample. The full loss for optimizing the model weights was computed as

3 $${{{\mathcal{L}}}}=0.1\times {{{{\mathcal{L}}}}}_{\rm{MSE}}+{{{{\mathcal{L}}}}}_{\rm{ED}}$$

Note that because both dense layers make use of the same information in the bottleneck layer, and because only one functional image is evaluated at a time, our model does not use or require any temporal information, autocorrelation or variance across images or movements to be trained on.

#### Decoding schemes

We implemented three decoding schemes differing in how the data were split into training and test sets. These decoding schemes are described in the following.

Within-participant decoding Here, we split the data of each participant into two equally sized partitions (50%/50% split). The model was trained on one-half of the data of all participants and then tested on the other half of the data of all participants. This cross-validation procedure allowed the model to learn about the intricacies and details of each participant’s MR signal and behaviors while still having to generalize across participants and to new data of the same participants (Extended Data Fig. 3).

Across-participant decoding To test whether the model generalizes to held-out and hence fully independent participants, we further implemented an across-participant decoding scheme. This scheme represents our default pipeline and was used to obtain the main results in Fig. 2. Each dataset was split into five equally sized partitions containing different participants. We then trained the model on four of these data partitions and then decoded from the fifth (80%/20% split). This procedure was cross-validated until all data partitions and hence all participants were tested once. The across-participant decoding scheme requires the model to generalize to eyeballs and behavioral priors that it has not encountered during training. The fMRI and eye-tracking data, however, have been acquired on the same scanner and with the same scanning protocol.

Across-dataset decoding Finally, we tested whether DeepMReye generalizes across datasets that have been acquired in independent participants performing different viewing tasks scanned on different scanners and with different scanning protocols. We trained the model in a leave-one-dataset-out fashion using all datasets, meaning that the model was trained on all datasets except one and then tested on the one that was held out. This procedure was cross-validated until all datasets and hence all participants were tested once. Note that the voxel sizes and repetition times used for the acquisition of the key datasets 1–5 were similar but that the model still had to generalize across different participants, MRI scanners and other scan parameters (for example, slice orientation). Interestingly, despite higher EE and lower *R*^2^ scores than within-dataset decoding (Extended Data Fig. 3), the across-dataset decoding scheme led to relatively high Pearson correlations. This indicates that the main reason for the lower performance scores is the scaling of the decoding output relative to the test labels, likely because the data range of the training and testing labels differed. Importantly, this also suggests that the presence of putative eye movements, but not their correct amplitude, could still be detected accurately, which is most important for many fMRI analyses or nuisance models. Further note that the model performance of the across-dataset procedure would likely further improve if even more diverse viewing behaviors and fMRI data were used for model training (Extended Data Fig. 3).

### Model quantification

To quantify model performance, we used the EE as described above for model training and evaluation. In addition, we computed the Pearson correlation and the *R*^2^ score as implemented in scikit-learn^40^ between real and decoded gaze path for model inference. The *R*^2^ score expresses the fraction of variance that our gaze decoding accounted for in the ground truth gaze path.

4 $${R}^{2}=1-\frac{\mathop{\sum }\nolimits_{i = 1}^{M}({y}_{i}-{\hat{y}}_{i})}{\mathop{\sum }\nolimits_{i = 1}^{M}({y}_{i}-\bar{y})}$$

Here, *y*_*i*_ is the ground truth of sample *i*, ${\hat{y}}_{i}$ is the predicted value and $\bar{y}$ is the mean value. Unlike the Pearson correlation, or the squared Pearson correlation, the *R*^2^ score used here is affected by the scaling of the data and can be arbitrarily negative. Model performance scores were calculated for vertical and horizontal coordinates and then averaged to obtain the final scores. Moreover, we computed the error as a FoS as the EE divided by the stimulus diagonal (that is, $\sqrt{X{\textrm{rang}}e^{2}+Y{\textrm{range}}^{2}}$).

Importantly, if a participant did not fixate at the cued locations or if there was noise in the ground truth eye-tracking data, that participant would likely show high error estimates even if our decoding worked perfectly. All model performance scores are therefore a function of the accuracy of our model as well as the accuracy of the test labels. The error estimates of the across-participant decoding scheme could therefore be used to monitor task compliance in the absence of camera-based eye tracking.

### Shuffling-based voxel-wise salience score

To test which voxels were most informative for our model for gaze decoding, we iteratively shuffled the time courses of the eye mask voxels and quantified how much this shuffling affected the EE of our across-participant decoding model. This influence is captured in the salience score of a voxel, which reflects the average increase in the EE caused by shuffling the time course of the respective voxel. To reduce computational costs, we did not shuffle individual voxels but entire slices, which were selected randomly for each iteration from one of the three dimensions (*XYZ*). We repeated this for each sample across all participants five times with an average of 10,640 shuffles per participant. Each voxel was included in multiple shuffles, and we averaged the resulting scores across iterations to obtain one final saliency score per voxel, which we then visualized for inspection (Supplementary Fig. 2).

### Decoding from the eyeballs and early visual cortex with time-shifted data

To investigate if the decoding is instantaneous or further improves when temporal delays are being considered, we shifted the functional image time series relative to the gaze labels. We again used the free-viewing dataset^15^, because it featured the most complex and natural viewing behaviors in our sample. For each image shift (0–10 TRs), we retrained the full model and tested it on held-out participants using the across-participant decoding scheme.

To further assess whether DeepMReye can also be used to decode from brain activity directly, we used the same temporal shifting procedure while decoding from area V1. The regions-of-interest mask was obtained by thresholding the Juelich atlas mask ‘Visual_hOc1.nii’ at 60% probability and reslicing it to the resolution of our template space (Supplementary Fig. 5). As the model is agnostic to the dimensions of the input data, decoding from region of interests other than the eyeballs required no change in model architecture.

### Effect of training set size

To evaluate how the number of participants in the training set influences decoding performance, we retrained the model using different subsets of participants across model iterations (1–21 participants). For each iteration, we tested the model on the same six participants, which were not part of the training set. To ensure that the results were robust and did not depend on individual details of single participants used for model training, we repeated this procedure five times for each training set size and then averaged the results. To do so, we randomly assigned participants to the training set in each cross-validation loop while keeping the test set fixed. Moreover, to avoid overfitting to these small training sets, we reduced the number of training epochs using *e* = 2 + *N*, with *N* being the number of participants in the current training run and *e* the number of epochs. Each epoch reflects a total of 12,000 samples (1,500 gradient steps × 8 samples) that were passed through the network during training. We kept the number of gradient steps in each epoch constant (*n* = 1,500).

In addition to testing how the number of participants in the training set influenced model performance, we further quantified how much the amount of data acquired for each one of the participants influenced model performance. To do so, we again subsampled the training data like before but this time subsampled the data within each participant in addition to the number of participants in the training set (*n* = 8 and *n* = 20). We trained the model using different proportions of the data of each participant (1, 5, 10, 20, 30, 40, 50, 60, 70, 80, 90 and 100% of the available data), which approximately corresponded to 0.3, 2, 4, 7, 11, 14, 18, 21, 25, 28, 32 and 35 average minutes of scanning time. The smallest training set we tested comprised only around 134 s worth of data (21 s per participant × 8 participants × 4/5 cross-validation splits). We again quantified model performance as the Pearson correlation and the *R*^2^ score between the real and the predicted gaze path of each held-out participant in the test set (Extended Data Fig. 7).

### Eyes-closed eye tracking

As a proof of concept, we tested whether DeepMReye is capable of decoding gaze position or rather the state of the eyeballs while the eyes are closed. We trained the model on the camera-based eye tracking labels of the four participants in dataset 6. We included the data acquired with all nine scanning protocols and with all viewing behaviors tested (fixation, smooth pursuit and picture viewing). We then evaluated the model on one participant, who was instructed to close their eyes and move them alternatingly from left to right or up and down. The participant indicated the direction of movement by pressing a button, which was used to color the coordinates in Fig. 3b. The participant performed this task nine times for 1 min each. To reduce overfitting to the viewing behaviors in the training set, we here used a higher dropout rate in the fully connected layers (drop ratio = 0.5) than in our default model (drop ratio = 0.1).

### Decoding at subimaging temporal resolution

Because different imaging slices are being acquired at different times, and because the MR signal of a voxel could be affected by eye motion within each TR, we tested whether our model is capable of decoding gaze position at subimaging temporal resolution. Across different model iterations, we retrained and retested the model using different numbers of gaze labels per TR (*n* = 1–10 labels), each time testing how much variance the decoded gaze path explained in the true gaze path. Decoding different numbers of gaze labels per TR was achieved by replicating the bottleneck layer *n* times, each one decoding gaze position for their respective time points using a fully connected layer. Importantly, the weights between these layers were not shared, which allowed each layer to use a different node in the bottleneck layer. Each layer could therefore capture unique information at its corresponding within-TR time point to decode its respective gaze label. To keep the overall explainable variance in the test set gaze path constant, we always upsampled the decoded gaze path to a resolution of ten labels per TR using linear interpolation before computing the *R*^2^ score for each model iteration. Potential differences in model performance across iterations can therefore not be explained by differences in explainable variance. These final test *R*^2^ scores were range normalized within each participant for visualization (Fig. 2f).

The subimaging labels were not specifically matched to the slice timing of the imaging sequence. However, because all imaging sequences featured simultaneous multislice/multiband acquisition, the eyes were included at every slice time in at least one slice. Future studies will need to estimate the subimaging resolution for sequential single-slice acquisitions, but because these often sample slices in an interleaved fashion, the eyes will still be featured at various time points throughout the TR. Intriguingly, exploring the influence of slice timing and other sequence parameters on the subimaging resolution may lead to sequences that further improve the accuracy and the temporal resolution of the subimaging decoding beyond the results presented here (Fig. 2f and Extended Data Fig. 8).

### Eyes-closed versus eyes-open classification

We tested if our model could decode whether the eyes were open or closed during the acquisition of a functional volume. To do so, we used the camera-based eye-tracking data acquired for the smooth pursuit dataset 4 (ref. ^14^) to compute the proportion of time spent with the eyes closed for each volume of each participant. We trained and tested our model on these labels using our across-participant decoding scheme again with a 80%/20% train/test cross-validation split. This procedure was iterated until all participants were included in the test set once. One value per volume was decoded. As the only loss function, we used the mean squared error between the real and predicted proportion of time spent with eyes closed. We obtained a continuous decoding time course for each participant in the test set by decoding the proportion of time spent with eyes closed from each volume. To quantify classification performance of the model, we thresholded this time series, for example, at the 10% level to obtain categorical labels (eyes open versus eyes closed). These binary labels reflected whether the eyes were open for the full time of volume acquisition or whether they were closed for more than, for example, 10% of the time. We then used these labels to calculate accuracy and balanced accuracy (that is, (true positive rate + true negative rate)/2) as well as to visualize receiver operating characteristic (ROC) curves (Extended Data Fig. 9). The results of all thresholds in steps of 10% are reported in the following (threshold–balanced accuracy): 0%–81.9%, 10%–84.6%, 20%–84.3%, 30%–81.8%, 40%–79.3%, 50%–78.7%, 60%–77.7%, 70%–77.8%, 80%–77.1%, 90%–74.7%.

### Functional imaging analyses

We tested whether the decoding output of DeepMReye is suitable for the analysis of functional imaging data by regressing it against brain activity using a mass-univariate GLM. This analysis was expected to uncover brain activity related to eye movements in visual, motion and oculomotor regions. To demonstrate that our approach is applicable even for natural and complex viewing behavior, we conducted these analyses on the visual search dataset^15^.

First, we decoded the median gaze position at each imaging volume using all cross-validation schemes described above. We then obtained an approximate measure of eye movement amplitude by computing the vector between gaze positions of subsequent volumes. Based on the vector length, or the amplitude of decoded putative eye movements, we built two regressors of interest, one for far eye movements (>66th percentile of movement amplitudes) and one for short eye movements (<33rd percentile of amplitudes). The midsection was excluded to separate the modeled events in time. The two resulting regressors per scanning run were binarized and convolved with the hemodynamic response function implemented in SPM12 using default settings. Head motion parameters obtained during preprocessing were added as nuisance regressors. Contrasting the resulting model weight between far and short eye movements yielded one *t*-statistics map per participant.

To test which brain areas signaled the difference between far and short eye movements, we normalized the *t*-map of each participant to MNI space and smoothed it with an isotropic Gaussian kernel of 6 mm (full-width half-maximum). The smoothed statistical maps were then used to compute an *F*-statistic on group level using SPM12. Moreover, to compare the results obtained with DeepMReye to the ones of conventional eye tracking, we repeated the imaging analysis described above using gaze positions obtained with a conventional camera-based eye tracker. The final *F*-statistics maps were warped onto the fsaverage Freesurfer template surface for visualization using Freesurfer (https://surfer.nmr.mgh.harvard.edu/).

### Statistics and reproducibility

Datasets 1–5 were used in previous reports and are described in detail in the original publications^11–15^ as well as in dedicated Methods sections. No statistical methods were used to predetermine the sample size of dataset 6, but no significance testing was performed. All datasets met the assumptions, and individual data distributions are shown in all figures. No randomization or blinding was used, and data exclusion criteria are described in the respective Methods sections of each dataset (for example, participants were excluded if the eyes were not scanned and were missing on the functional images).

### Reporting Summary

Further information on research design is available in the Nature Research Reporting Summary linked to this article.

## Online content

Any methods, additional references, Nature Research reporting summaries, source data, extended data, supplementary information, acknowledgements, peer review information; details of author contributions and competing interests; and statements of data and code availability are available at 10.1038/s41593-021-00947-w.

## Supplementary information

Supplementary Information Supplementary Figs. 1–5 and Tables 1 and 2.

Reporting Summary

## Data Availability

We analyzed data of multiple previous reports, which can be requested from the respective authors. Dataset 1 is part of a larger data-sharing initiative and can be downloaded at http://fcon_1000.projects.nitrc.org. We further share online on Open Science Framework (10.17605/OSF.IO/MRHK9) exemplary data to illustrate our pipeline (also see ‘Code availability’ statement) as well as the source data for Figs. 1–4 and Extended Data Figs. 1–10. Moreover, we share pretrained model weights estimated on the datasets used in the present work. These model weights allow for decoding of viewing behavior without retraining the model in certain scenarios (see online documentation for more details at https://github.com/DeepMReye). Source data are provided with this paper.

## Source data

Source Data Fig. 1 Multivoxel pattern of the eyeballs.
Source Data Fig. 2 Statistical source data.
Source Data Fig. 3 Statistical source data.
Source Data Fig. 4 Unthresholded statistical maps.
Source Data Extended Data Fig. 1 Statistical source data.
Source Data Extended Data Fig. 2 Statistical source data.
Source Data Extended Data Fig. 3 Statistical source data.
Source Data Extended Data Fig. 4 Statistical source data.
Source Data Extended Data Fig. 5 Statistical source data.
Source Data Extended Data Fig. 6 Statistical source data.
Source Data Extended Data Fig. 7 Statistical source data.
Source Data Extended Data Fig. 8 Statistical source data.
Source Data Extended Data Fig. 9 Statistical source data.
Source Data Extended Data Fig. 10 Unthresholded statistical maps.

## Code availability

The DeepMReye model code can be found on GitHub (https://github.com/DeepMReye), along with user documentation and a frequently asked questions page. Moreover, we share Colab notebooks that illustrate the use of DeepMReye using exemplary data. Finally, we share exemplary eye-tracking calibration scripts that can be easily adapted to acquire training data for DeepMReye.
