# Author Correction: Magnetic resonance-based eye tracking using deep neural networks

**DOI:** 10.1038/s41593-023-01322-7

**Published:** 2023-04-12

**Authors:** Markus Frey, Matthias Nau, Christian F. Doeller

**Affiliations:** 1 Kavli Institute for Systems Neuroscience, Centre for Neural Computation, The Egil and Pauline Braathen and Fred Kavli Centre for Cortical Microcircuits, Jebsen Centre for Alzheimer’s Disease, Norwegian University of Science and Technology, Trondheim, Norway; 2 Max Planck Institute for Human Cognitive and Brain Sciences, Leipzig, Germany; 3 Institute of Psychology, Leipzig University, Leipzig, Germany

**Keywords:** Cognitive neuroscience, Visual system, Oculomotor system, Computational neuroscience

Correction to: *Nature Neuroscience* 10.1038/s41593-021-00947-w. Published online 8 November 2021.

This paper was originally published under a standard Springer Nature license (© The Author(s), under exclusive licence to Springer Nature America, Inc.). It is now available as an open-access paper under a Creative Commons Attribution 4.0 International license, © The Author(s). The error has been corrected in the print, HTML and PDF versions of the article.

