# Supplementary material for: Magnetic resonance-based eye tracking using deep neural networks
Source: Nat Neurosci. 2021 Nov 8;24(12):1772–9. doi: 10.1038/s41593-021-00947-w (PMC10097595; doi:10.1038/s41593-021-00947-w)
Supplement: Supplementary file 1 — Supplementary Figs. 1–5 and Tables 1 and 2. [file 41593_2021_947_MOESM1_ESM.pdf]

nature portfolio

https://doi.org/10.1038/s41593-021-00947-w

Supplementary information

Magnetic resonance-based eye tracking using deep neural networks

In the format provided by the
authors and unedited

Supplementary Figures

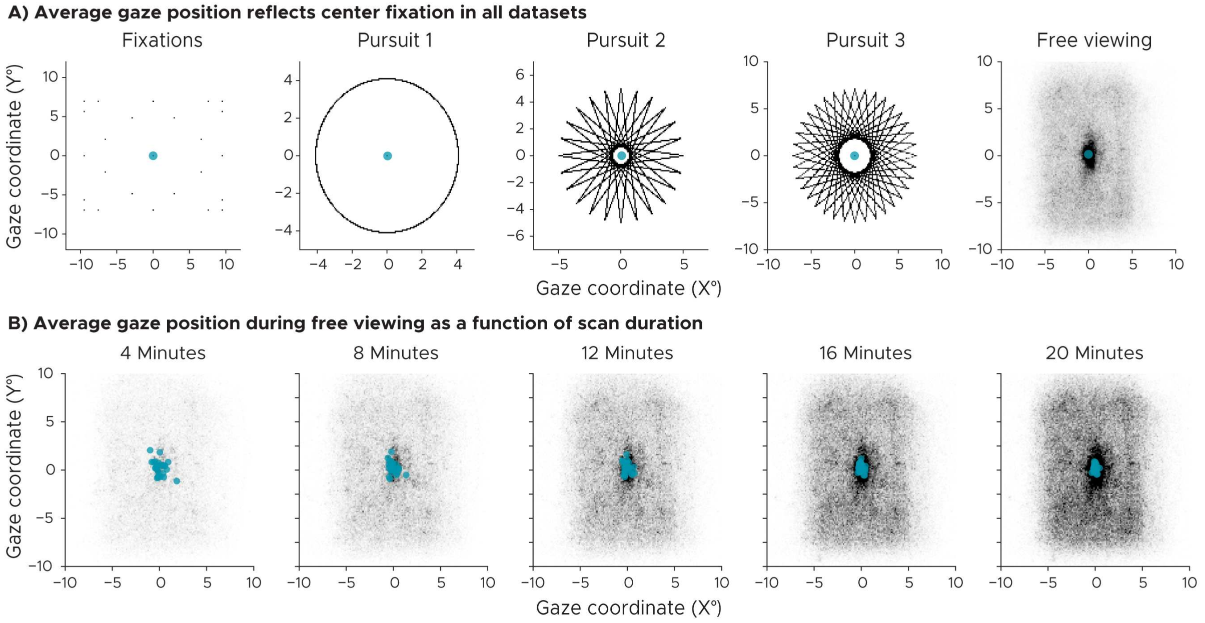

Supplementary Figure 1: Average gaze coordinate reflects center fixation. A) Average gaze coordinate for the main datasets tested. We plot 2D histograms of the sampled gaze positions averaged across participants within each dataset. Darker colors depict higher occupancy. In addition, we plot the average gaze position of each dataset and participants (blue dot). B) Average gaze position as a function of scan duration in the free-viewing dataset. We again plot 2D histograms similar to (A) for various scan durations (4,8,12,16,20 minutes), as well as the average gaze position of each participant (blue dots). Color scale was normalized relative to the 20 minutes histogram. Note that in all cases the average gaze positions clustered around the center of the respective distributions.

Correspondence: markus.frey@ntnu.no & matthias.nau@nih.gov

Shuffling-based voxel-wise saliency score for the decoding of gaze position

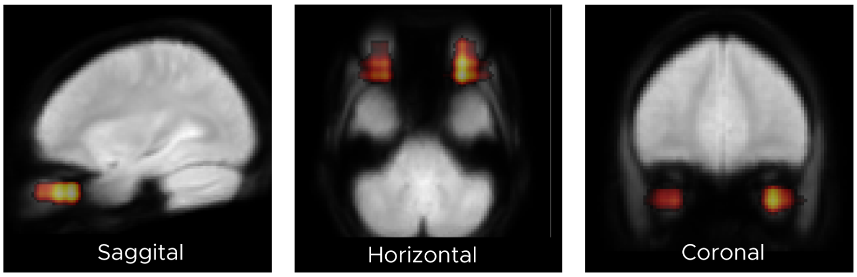

Thresholded at top 10% most salient voxels within the eye mask

Supplementary Figure 2: Gaze decoding relies most strongly on voxels that overlap with the eyeballs and optic nerves. We plot the saliency score for each voxel obtained via time-course shuffling. It expresses how strongly shuffling the time course of a voxel influenced the Euclidean Error of our model. We depict the across-participant average saliency scores for the top 10% most salient voxels in the free-viewing dataset overlaid on our co-registration group template. Hotter colors depict higher salience scores.

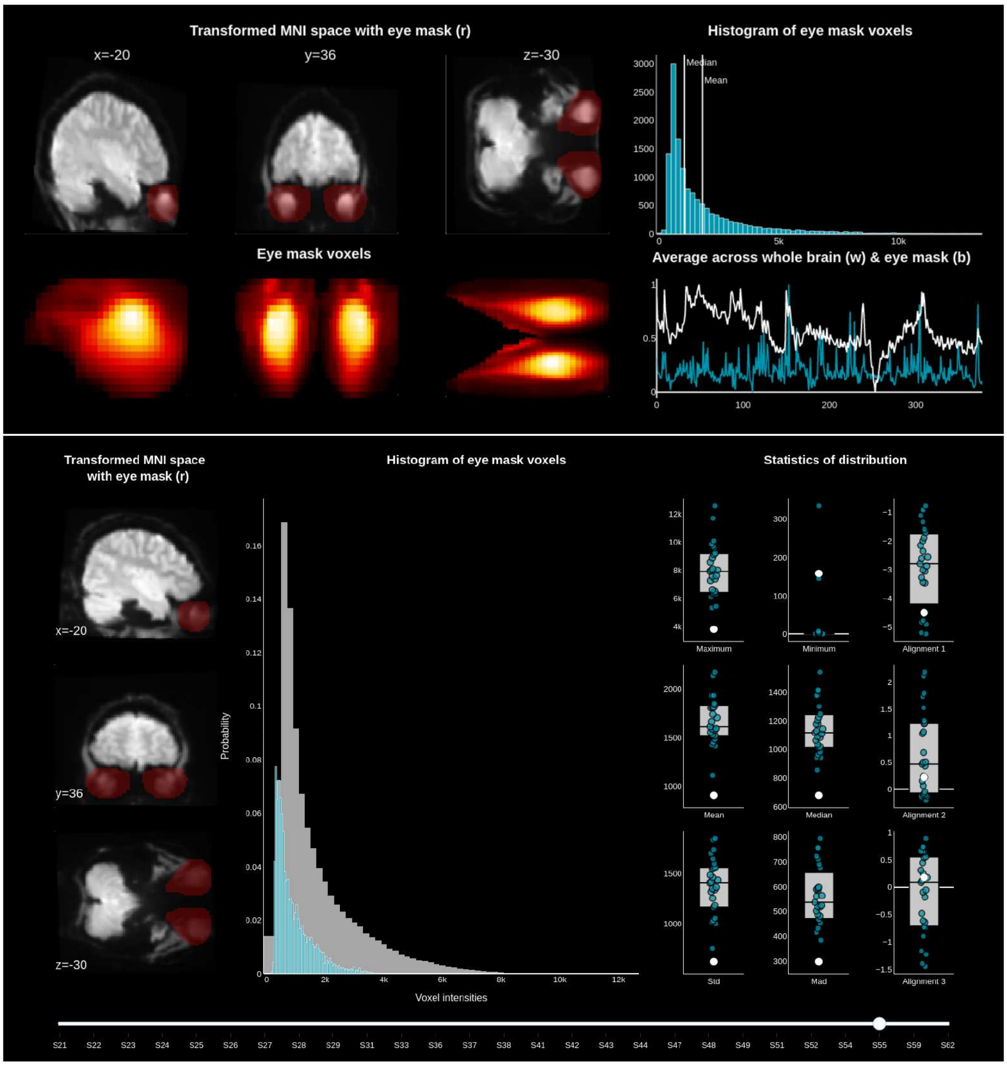

Supplementary Figure 3: Quality-check reports generated by DeepMReye. (Top) Within-participant report. Our pipeline automatically saves this interactive figure as an HTML-file for each participant after eyeball coregistration. It shows the transformed-voxel intensities across the whole brain as well as the co-registered eye mask (top left panel). Bottom left panel zooms in on the eye-mask voxels, top right panel shows a histogram of these voxel values for visual inspection. Bottom right panel shows the average time course of all voxels and of the eye-mask voxels. (Bottom) Across-participants report. Our pipeline automatically saves this interactive figure as an html-file for all participants after eyeball coregistration. Users can adjust the slider on the bottom to navigate through the data of all participants. Left panel: average EPI image and eye masks (red) after coregistration. Middle panel: histogram of voxel intensities averaged across all participants (grey) as well as for the currently selected participant (blue). Right panel: descriptive statistics of distribution across all participants (blue dots) with selected participant highlighted (white dot) and group-level Whisker-box-plots for the histogram maximum, minimum, mean, median, standard deviation, median absolute deviation, average coregistration (warping) parameters for head, facial bounding box and eyeballs. Note that this figure purposefully shows an example outlier participant, who seems to have very little and distorted signal in the area around the eyes, which is reflected in the histogram statistics of this participant.

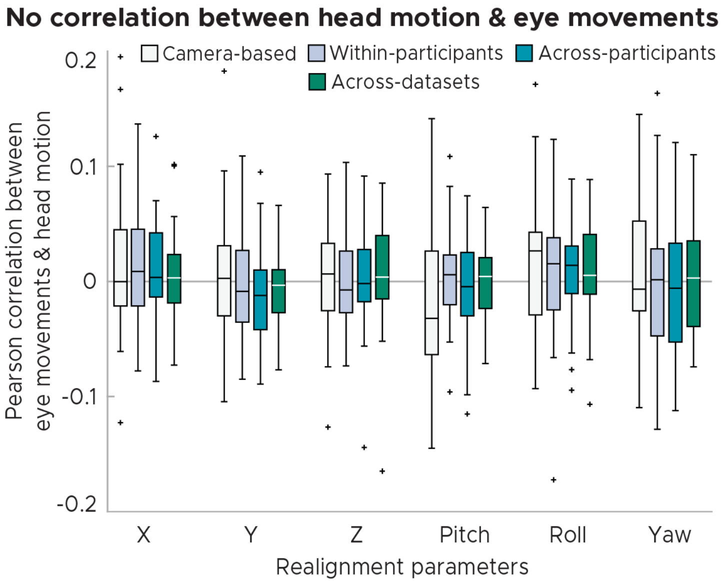

Supplementary Figure 4: No correlation between eye movements and head motion in visual search dataset 5. Eye movements were computed as the vector length between gaze positions of subsequent volumes. Head motion estimates reflect the 6 SPM12-realignment parameters. We plot Whisker-box-plots (central line: median, box: 25th and 75th percentile, whisker: all data points not considered outliers, outliers: data points outside 1.5x interquartile range) of this correlation computed for gaze labels obtained with camera-based eye tracking as well as with three cross-validation schemes of DeepMReye (within-participant-, across-participant- and across-dataset prediction).

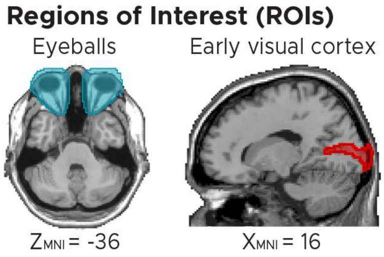

Supplementary Figure 5: Visualisation of eyeball and visual cortex (V1) masks used for decoding in Figure 2E. Eyeballs were manually segmented in the structural scan of the SPM-template participant "Colin27". The V1 mask was obtained by thresholding the Juelich-atlas mask "Visual_hOc1.nii" at 60 percent probability. MNI coordinates added. For decoding, both masks were resliced to 2mm isotropic to match the voxel resolution of our template space.

Supplementary Tables

Dataset	Behavior	TR	Voxel size	Participants	#TRs	#Minutes	Gaze labels	Field of View
1	Fixation	800ms	$2.4mm^3$	n=170	270	3.60	Target	19° x 15°
2	Pursuit	870ms	$2.0mm^3$	n=9	2778	40.28	Target	8° x 8°
3	Pursuit	1020ms	$2.0mm^3$	n=34	3568	60.65	Both	10° x 10°
4	Pursuit	1000ms	$2.0mm^3$	n=24	3961	66.01	Both	15° x 15°
5	Free viewing	1000ms	$2.0mm^3$	n=27	2128	35.46	Camera	17° x 17°
		1250ms	$1.5mm^3$		287			
		1800ms	$1.5mm^3$		200			
		2500ms	$1.5mm^3$		144			
		1250ms	$2.0mm^3$		287			
6	All the above	1800ms	$2.0mm^3$	n=4	200	~ 6	Both	30° x 15°
		2500ms	$2.0mm^3$		144			
		1250ms	$2.5mm^3$		287			
		1800ms	$2.5mm^3$		199			
		2500ms	$2.5mm^3$		144			

Supplementary Table 1: Overview of the six datasets. We list the dataset number, the viewing behavior that was tested, the repetition time (TR) and voxel size of the imaging protocol, the number of participants, the amount of data acquired for each participant expressed as the average number of acquired volumes (#TRs) and as the total scanning time (#Minutes), the type of gaze labels that DeepMReye was trained and tested on (incl. camera-based labels, screen coordinates of the fixation target, or both) as well as the task-relevant field of view (FoV) of the participant.

Correspondence: markus.frey@ntnu.no & matthias.nau@nih.gov

Sequence	Voxel size	TR	TE	FA	MB	pF	#Slices
1	$1.5mm^3$	1250ms	26ms	66	4	7/8	40
2	$1.5mm^3$	1800ms	26ms	74	4	7/8	40
3	$1.5mm^3$	2500ms	26ms	80	4	7/8	40
4	$2.0mm^3$	1250ms	26ms	66	4	7/8	60
5	$2.0mm^3$	1800ms	26ms	74	4	7/8	60
6	$2.0mm^3$	2500ms	26ms	80	4	7/8	60
7	$2.5mm^3$	1250ms	26ms	66	4	7/8	60
8	$2.5mm^3$	1800ms	26ms	74	4	7/8	60
9	$2.5mm^3$	2500ms	26ms	80	4	7/8	60

Supplementary Table 2: Sequence parameters of the 9 EPI-protocols used in the acquisition of dataset 6. For each sequence, we list the isotropic voxel size, the repetition time (TR), the echo time (TE), the flip angle (FA), the multiband factor (MB), partial Fourier factor (pF) and the number of slices (#Slices). All flip angles were aligned to the respective Ernst angle.
