# Supplementary material for: Magnetic resonance-based eye tracking using deep neural networks
Source: Nat Neurosci. 2021 Nov 8;24(12):1772–9. doi: 10.1038/s41593-021-00947-w (PMC10097595; doi:10.1038/s41593-021-00947-w)
Supplement: Supplementary file 2 — Reporting Summary [file 41593_2021_947_MOESM2_ESM.pdf]

nature portfolio

Corresponding author(s): Markus Frey, Matthias Nau

Last updated by author(s): Sep 1, 2021

Reporting Summary

Nature Portfolio wishes to improve the reproducibility of the work that we publish. This form provides structure for consistency and transparency in reporting. For further information on Nature Portfolio policies, see our Editorial Policies and the Editorial Policy Checklist.

Statistics

For all statistical analyses, confirm that the following items are present in the figure legend, table legend, main text, or Methods section.

n/a	Confirmed	
☐	☒	The exact sample size (*n*) for each experimental group/condition, given as a discrete number and unit of measurement
☐	☒	A statement on whether measurements were taken from distinct samples or whether the same sample was measured repeatedly
☒	☐	The statistical test(s) used AND whether they are one- or two-sided<br>*Only common tests should be described solely by name; describe more complex techniques in the Methods section.*
☒	☐	A description of all covariates tested
☐	☒	A description of any assumptions or corrections, such as tests of normality and adjustment for multiple comparisons
☐	☒	A full description of the statistical parameters including central tendency (e.g. means) or other basic estimates (e.g. regression coefficient) AND variation (e.g. standard deviation) or associated estimates of uncertainty (e.g. confidence intervals)
☒	☐	For null hypothesis testing, the test statistic (e.g. *F*, *t*, *r*) with confidence intervals, effect sizes, degrees of freedom and *P* value noted<br>*Give P values as exact values whenever suitable.*
☒	☐	For Bayesian analysis, information on the choice of priors and Markov chain Monte Carlo settings
☒	☐	For hierarchical and complex designs, identification of the appropriate level for tests and full reporting of outcomes
☐	☒	Estimates of effect sizes (e.g. Cohen's *d*, Pearson's *r*), indicating how they were calculated

Our web collection on statistics for biologists contains articles on many of the points above.

Software and code

Policy information about availability of computer code

Data collection	Matlab (2019b), Psychtoolbox (3.0)
Data analysis	Matlab (2019b), Python (3.7), tensorflow-gpu (2.2.0), numpy (1.19.1), pandas (1.0.5), matplotlib (3.2.2), scipy (1.5.0), ipython(7.13.0), plotly (4.14.3)

For manuscripts utilizing custom algorithms or software that are central to the research but not yet described in published literature, software must be made available to editors and reviewers. We strongly encourage code deposition in a community repository (e.g. GitHub). See the Nature Portfolio guidelines for submitting code & software for further information.

Data

Policy information about availability of data

All manuscripts must include a data availability statement. This statement should provide the following information, where applicable:

- Accession codes, unique identifiers, or web links for publicly available datasets
- A description of any restrictions on data availability
- For clinical datasets or third party data, please ensure that the statement adheres to our policy

We analyzed data of multiple previous reports, which are available from the respective authors upon request. Dataset 1 is part of a larger data-sharing initiative and can be downloaded here: http://fcon_1000.projects.nitrc.org. We share online exemplary data to illustrate our pipeline (also see "Code availability" statement), as well as pre-trained model weights estimated on the datasets used in the present work. These model weights allow decoding viewing behavior without re-training the model in certain scenarios (see online documentation for more details: https://github.com/DeepMReye)

March 2021

Field-specific reporting

Please select the one below that is the best fit for your research. If you are not sure, read the appropriate sections before making your selection.

☒ Life sciences ☐ Behavioural & social sciences ☐ Ecological, evolutionary & environmental sciences

For a reference copy of the document with all sections, see nature.com/documents/nr-reporting-summary-flat.pdf

Life sciences study design

All studies must disclose on these points even when the disclosure is negative.

Sample size	We tested 6 independent datasets in this study:<br>Fixation dataset: 170 Participants<br>Pursuit 1: dataset 24 Participants<br>Pursuit 2 dataset: 34 Participants<br>Pursuit 3 dataset: 9 Participants<br>Free viewing dataset: 27 participants<br>Dataset 6: 4 Participants<br><br>The number of datasets was chosen to include the following viewing behaviors (free viewing, fixation, smooth pursuit, eyes-closed)
Data exclusions	Free viewing dataset: We excluded 9 participants because the functional images did not or only partially include the eyeballs<br>Smooth pursuit dataset 3: 5 participants where excluded as no eye tracking data has been obtained during the experiment<br>Fixation dataset: This dataset was limited to participants with no visible motion artifcats in either the T1- or the average T2 weighted images and the eyeballs were fully included in the functional images
Replication	All effects were replicated in 6 independent datasets
Randomization	We cross validated all experiments, with the data split into 5 distinct groups each containing 1/5th of all subjects. This procedure was iterated until each split was used as test set once.
Blinding	For dataset 1-5 see blinding information in original manuscripts. For dataset 6 no blinding was used as all four participants performed all tasks.

Reporting for specific materials, systems and methods

We require information from authors about some types of materials, experimental systems and methods used in many studies. Here, indicate whether each material, system or method listed is relevant to your study. If you are not sure if a list item applies to your research, read the appropriate section before selecting a response.

Materials & experimental systems

n/a	Involved in the study	
☒	☐	Antibodies
☒	☐	Eukaryotic cell lines
☒	☐	Palaeontology and archaeology
☒	☐	Animals and other organisms
☐	☒	Human research participants
☒	☐	Clinical data
☒	☐	Dual use research of concern

Methods

n/a	Involved in the study	
☒	☐	ChIP-seq
☒	☐	Flow cytometry
☐	☒	MRI-based neuroimaging

Human research participants

Policy information about studies involving human research participants

Population characteristics	Datasets 1-5 were part of previous reports and are described in dedicated sections (see "Methods") and in detail in the original publications (Alexander et al. 2017, Nau et al. 2018, Nau et al. 2018, Polti et al. 2021, Julian et al. 2018). The sixth dataset included four male participants (Age 25-32).
Recruitment	Datasets 1-5 were part of previous reports and recruitment details can be found in the original publications. Dataset 6 was recruited through local recruitment at the University.
Ethics oversight	Data acquisition was approved by the regional committees for medical and health research ethics (REK sør-øst), Norway, and participants gave written informed consent prior to scanning. Ethics oversight for Datasets 1-5 can be found in the original publications

Note that full information on the approval of the study protocol must also be provided in the manuscript.

Magnetic resonance imaging

Experimental design

Design type: Event-related

Design specifications: Different experimental designs for each dataset, but DeepMReye considers each functional volume as separate input.
The following number of volumes were used per participant.
Dataset 1: 270
Dataset 2: 3961
Dataset 3: 3568
Dataset 4: 2778
Dataset 5: 2128
Dataset 6: 287, 200 or 144 depending on the fMRI sequence used

Behavioral performance measures: Four datasets recorded additional eye tracking data

Acquisition

Imaging type(s): functional

Field strength: 3T

Sequence & imaging parameters: The data were acquired using 14 scan protocols described in detail in the methods and the original publications cited in the manuscript.

Area of acquisition: We used region-of-interest masks for the eyeballs (manual segmentation) and the early visual cortex (Juelich Atlas)

Diffusion MRI ☐ Used ☒ Not used

Preprocessing

Preprocessing software: Matlab, SPM12, ANTs

Normalization: All data were normalized to MNI-template and a group average template using SPM12 and ANTs

Normalization template: MNI-template and group-average template

Noise and artifact removal: Images were corrected for head motion and field distortions using SPM12

Volume censoring: No volume censoring was performed

Statistical modeling & inference

Model type and settings: DeepMReye is a convolutional neural network that uses three-dimensional data to classify a two-dimensional output; the horizontal (X) and vertical (Y) gaze coordinates on the screen. The model uses the voxel intensities from the eye masks as input and passes it through a series of 3D-convolutional layers interleaved with group normalization and non-linear activation functions (mish). In detail, the eye mask (input layer) is connected to a 3D convolutional block with a kernel size of 3 and strides of 1, followed by dropout and a 3D convolutional downsampling block which consists of one 3D-convolution followed by a 2x2x2 average pooling layer. After this layer, we use a total of six residuals blocks, in which the residual connection consists of one 3D convolutional block, concatenated via simple addition. Each residual block consists of group normalization, non-linear activation, and a 3D convolution, which is applied twice before being added to the residual connection. This results in a bottleneck layer consisting of 7680 units, which we resample to achieve sub-TR resolution.

Effect(s) tested: The first dense layer learns to decode gaze position by minimizing the Euclidean error between the predicted gaze position and the ground truth gaze position as a loss function. The second dense layer in turn predicts the Euclidean error used to train the first dense layer.

Specify type of analysis: ☐ Whole brain ☐ ROI-based ☒ Both

Anatomical location(s): The V1 mask was obtained by thresholding the Juelich-atlas mask at 60 percent probability. Eye masks were created by manually segmenting the eyeballs including the adjacent optic nerve, fatty tissue and muscle area in the Colin27 structural MNI template using itkSNAP

Statistic type for inference
(See Eklund et al. 2016)

voxel-wise

Correction

FWE

Models & analysis

n/a	Involved in the study
☒	☐ Functional and/or effective connectivity
☒	☐ Graph analysis
☐	☒ Multivariate modeling or predictive analysis

Multivariate modeling and predictive analysis

To quantify model performance, we used the Euclidean error for model training and evaluation. In addition, we computed the Pearson correlation and the R2-score as implemented in scikit-learn between real and decoded gaze path for model inference. The R2-score expresses the fraction-of-variance that our gaze decoding accounted for in the ground truth gaze path.
